# Conserved and repetitive motifs in an intrinsically disordered protein drive ⍺-carboxysome assembly

**DOI:** 10.1016/j.jbc.2024.107532

**Published:** 2024-07-04

**Authors:** Julia B. Turnšek, Luke M. Oltrogge, David F. Savage

**Affiliations:** 1 Department of Molecular and Cell Biology, University of California, Berkeley, California, USA; 2 Howard Hughes Medical Institute, University of California, Berkeley, California, USA; 3 Innovative Genomics Institute, University of California, Berkeley, California, USA

**Keywords:** carboxysome, intrinsically disordered protein, protein self-assembly, protein motif, cyanobacteria, bacterial microcompartment, phase separation, biological condensate

## Abstract

All cyanobacteria and some chemoautotrophic bacteria fix CO_2_ into sugars using specialized proteinaceous compartments called carboxysomes. Carboxysomes enclose the enzymes Rubisco and carbonic anhydrase inside a layer of shell proteins to increase the CO_2_ concentration for efficient carbon fixation by Rubisco. In the ⍺-carboxysome lineage, a disordered and highly repetitive protein named CsoS2 is essential for carboxysome formation and function. Without it, the bacteria require high CO_2_ to grow. How does a protein predicted to be lacking structure serve as the architectural scaffold for such a vital cellular compartment? In this study, we identify key residues present in the repeats of CsoS2, VTG and Y, which are necessary for building functional ⍺-carboxysomes *in vivo*. These highly conserved and repetitive residues contribute to the multivalent binding interaction and phase separation behavior between CsoS2 and shell proteins. We also demonstrate 3-component reconstitution of CsoS2, Rubisco, and shell proteins into spherical condensates and show the utility of reconstitution as a biochemical tool to study carboxysome biogenesis. The precise self-assembly of thousands of proteins is crucial for carboxysome formation, and understanding this process could enable their use in alternative biological hosts or industrial processes as effective tools to fix carbon.

Carboxysomes are proteinaceous cellular microcompartments that are the metabolic centerpieces of the bacterial CO_2_-concentrating mechanism. Each structure is >100 nm in diameter and encloses the enzymes carbonic anhydrase and Rubisco in a polyhedral-like shell, raising the luminal CO_2_ concentration and driving Rubisco to operate at its maximum rate and specificity (1, 2, 3, 4). There are two carboxysomal lineages that evolved convergently: ⍺-carboxysomes, which emerged in proteobacteria and were horizontally transferred to ⍺-cyanobacteria, and β-carboxysomes, which originated in β-cyanobacteria (5). In this work, we focus on the ⍺-carboxysomal lineage, using the proteobacterium *Halothiobacillus neapolitanus* as our model system (6, 7, 8, 9).

All carboxysomes require five essential protein components: Rubisco, carbonic anhydrase, hexameric shell proteins, pentameric shell proteins, and a scaffold protein. Much is known about how the enzymatic and shell proteins function in the metabolism and structure of the carboxysome (3, 5, 10, 11), as well as how the β-carboxysome scaffolding protein CcmM drives carboxysome biogenesis within the β-lineage (12, 13, 14, 15). Although both carboxysome lineages contain scaffolding proteins, these proteins are related in function alone; they have no sequence or structural similarity. In contrast to the β-lineage, how the ⍺-carboxysome scaffolding protein directs ⍺-carboxysome assembly is far less understood.

The ⍺-carboxysome scaffolding protein, CsoS2, is highly conserved and is required for carboxysome assembly. CsoS2 knockouts cannot produce carboxysomes, rendering the bacteria incapable of growing at atmospheric CO_2_ levels (0.04% CO_2_) (Fig. S1) (16, 17). CsoS2 is a large ∼90 kDa protein with three distinct domains (Fig. 1*A*) (18). The N-terminal domain (NTD) contains four alpha-helical repeats that bind Rubisco in a low-affinity and multivalent manner (19). The middle region (MR) has seven distinct repeats, followed by the C-terminal domain (CTD) with two repeats and a highly conserved C-terminal peptide.

**Figure 1:**
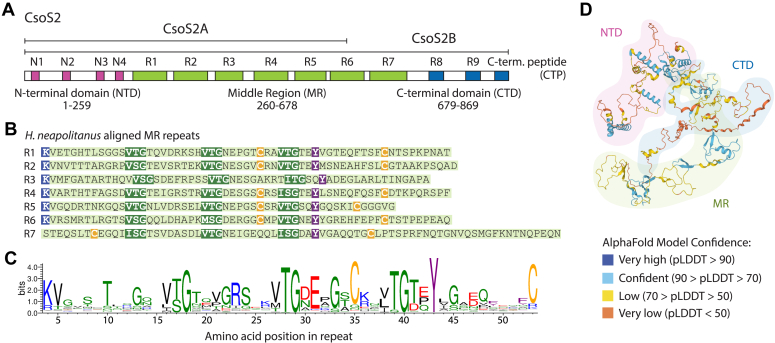
**CsoS2 middle region contains highly conserved and repeated motifs.***A*, domain architecture of CsoS2. Repeats within domains are indicated by colored blocks. Amino acid numbering is specific to *Halothiobacillus neapolitanus* CsoS2. *B*, alignment of all MR repeats in *H. neapolitanus* CsoS2, with highly conserved motifs highlighted. *C*, sequence logo of the MR repeat generated from an alignment of 1662 MR repeats identified across 272 dereplicated CsoS2 sequences. *Blue* is basic, *red* is acidic, *green* is polar/small, *black* is hydrophobic, *yellow* is cysteine, *purple* is aromatic. *D*, AlphaFold model of *H. neapolitanus* CsoS2 (UniProt O85041). pLDDT is AlphaFold’s per-residue confidence score, which scales from 0 to 100.

The CTD of CsoS2 binds to shell proteins and has been successfully used as an encapsulation peptide for heterologous cargo (16, 20, 21). Recent cryo-EM studies of mini-carboxysomes and the *Prochlorococcus* ⍺-carboxysome resolved CTD density, revealing how it spans shell–shell interfaces and modulates shell interactions, as well as overall carboxysome curvature and T number (22, 23). These structures show that the CTD exclusively binds to regions close to the pentamer interface, suggesting an importance for pentamer recruitment and binding. Interestingly, CsoS2 has a short (CsoS2A) and long (CsoS2B) form produced by a ribosomal frameshifting site in the sixth MR repeat, effectively cutting off the CTD in the short form (24). While the long form is essential for carboxysome formation, the short form is not (25). In *H. neapolitanus*, CsoS2A and B are found in equimolar ratios within the carboxysome (16, 26).

Less well studied is the MR domain, which makes up almost 50% of the CsoS2 sequence. The seven MR repeats have several intriguing highly conserved residues and sequence motifs. These were described previously (16, 27) and remain prominent in an up-to-date consensus sequence compiled from 272 de-replicated CsoS2 sequences in which each MR repeat was classified, extracted, and re-aligned against all other individual repeats. Four residues and motifs stand out in particular: (1) (V/I)(T/S)G triplets spaced eight amino acids apart (hereafter VTG repeats), (2) cysteine pairs, (3) a highly conserved lysine, and (4) a highly conserved tyrosine (Fig. 1, *B* and *C*).

In addition to this repeated motif structure, CsoS2 has a number of intrinsically disordered regions as identified by computational disorder predictors and corroborated by circular dichroism spectroscopy (19). AlphaFold of CsoS2 yields disordered coils and low confidence scores in the MR and CTD regions, though it accurately depicts the known NTD alpha helices (Fig. 1*D*) (28). The MR had not been resolved by either cryoEM or cryo-electron tomography (22, 29, 30) until very recently (while this work was in review) (23).

This work contributes to our understanding of how an intrinsically disordered protein (IDP) directs the assembly of the ⍺-carboxysome and the role of the MR's highly conserved residues. We show that some, but not all, of these residues are essential for the growth of *H. neapolitanus* in air. These residues bind to shell proteins in a weak yet highly multivalent fashion and also facilitate the formation of biological condensates when mixed with shell *in vitro*, which may mimic *in vivo* assembly. We found that three-component mixing of shell, Rubisco, and CsoS2 leads to the most robust condensate formation, driven primarily by the shell–CsoS2 interaction. With the addition of reductant, shell becomes more mobile in condensates, suggesting a redox-modulated assembly strategy in ⍺-carboxysomes.

## Results

### Identification of highly conserved motifs in the CsoS2 MR that are essential for carboxysome assembly

To probe the function of the highly conserved motifs in the MR repeats, we mutated these residues and assayed the growth of *H. neapolitanus* in air. *H. neapolitanus* needs carboxysomes to grow in air (0.04% CO_2_), but not at higher CO_2_ concentrations, enabling a selection system for deleterious CsoS2 variants. Because the MR has seven repeats and binding may display complicated behavior, a series of mutants was generated until the entire MR was disrupted (Fig. S2). VTGs were mutated to AAA, Y to A, K to A, and C to S. All strains were generated by knocking out the genomic copy of CsoS2 and re-inserting a complement or mutated copy into a neutral site on the genome (Fig. S1). All strains expressed similar amounts of CsoS2 (Fig. S3), though it should be noted that only CsoS2B was detected; it is likely that expression from the neutral site instead of the native operon reduced ribosomal frameshifting responsible for the production of nonessential CsoS2A.

**Figure 2:**
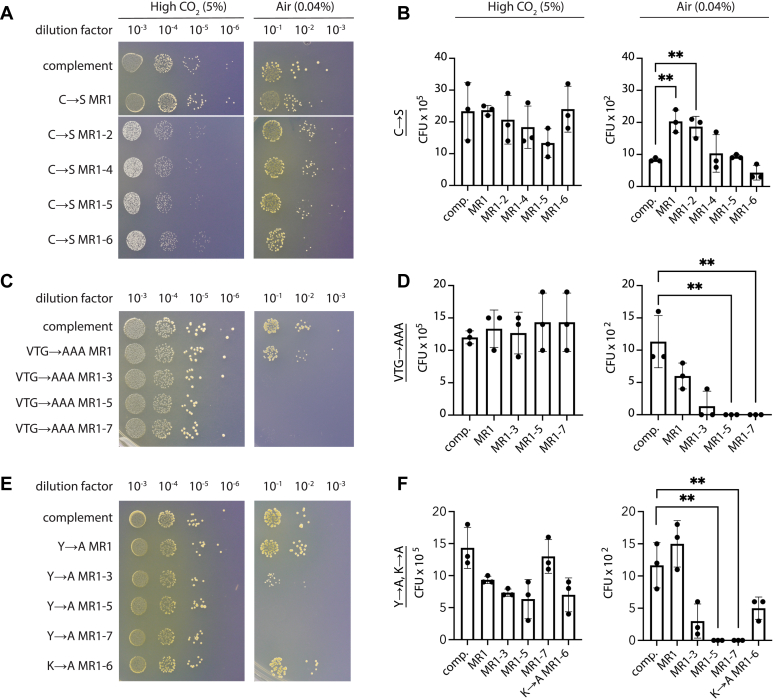
**VTG and Y mutations significantly affect the growth of *Halothiobacillus neapolitanus* in air.***A*, *C*, and *E*, representative plates from dilution spotting assays of CsoS2 mutants in *H. neapolitanus*, grown in high CO_2_ (5%) and air (0.04%). *B*, *D*, and *F*,) quantification of spotting assay results. Significance of ∗∗ is *p* ≤ 0.01 in an unpaired *t* test. Error bars show the SD of three replicate spotting plates. *A* and *B*, C→S mutants, (*C* and *D*) VTG→AAA mutants, and (*E* and *F*) Y→A and K→A mutants. The *white* splice line in (*A*) indicates that this image is a compilation of four plates. CFU, colony forming units; Comp, complement; MR, Middle region.

CsoS2 cysteine and lysine deletion strains showed no loss of growth in air (Fig. 2, *A*, *B*, *E*, and *F*), while VTG and tyrosine mutants showed a dramatic loss of growth (Fig. 2, *C*, *D*, *E*, and *F*). Growth was dependent on the number of VTG or Y motifs mutated, showing greater attenuation with more mutated repeats. It was unexpected to see no defect for the cysteine mutants, due to the purported role of redox in carboxysome formation (13, 18) and the seemingly obvious disulfide-bonding function of the conserved cysteine pairs. Despite robust growth of the CsoS2 cysteine mutant strains of *H. neapolitanus*, CsoS2 cysteine mutant carboxysomes could not be purified from *Escherichia coli* (Fig. S4), suggesting that cysteines play a structural role that strengthens the overall integrity of the complex but are not necessary for assembly or function *in vivo* under laboratory conditions.

### The CsoS2 MR binds to shell proteins

**Figure 3:**
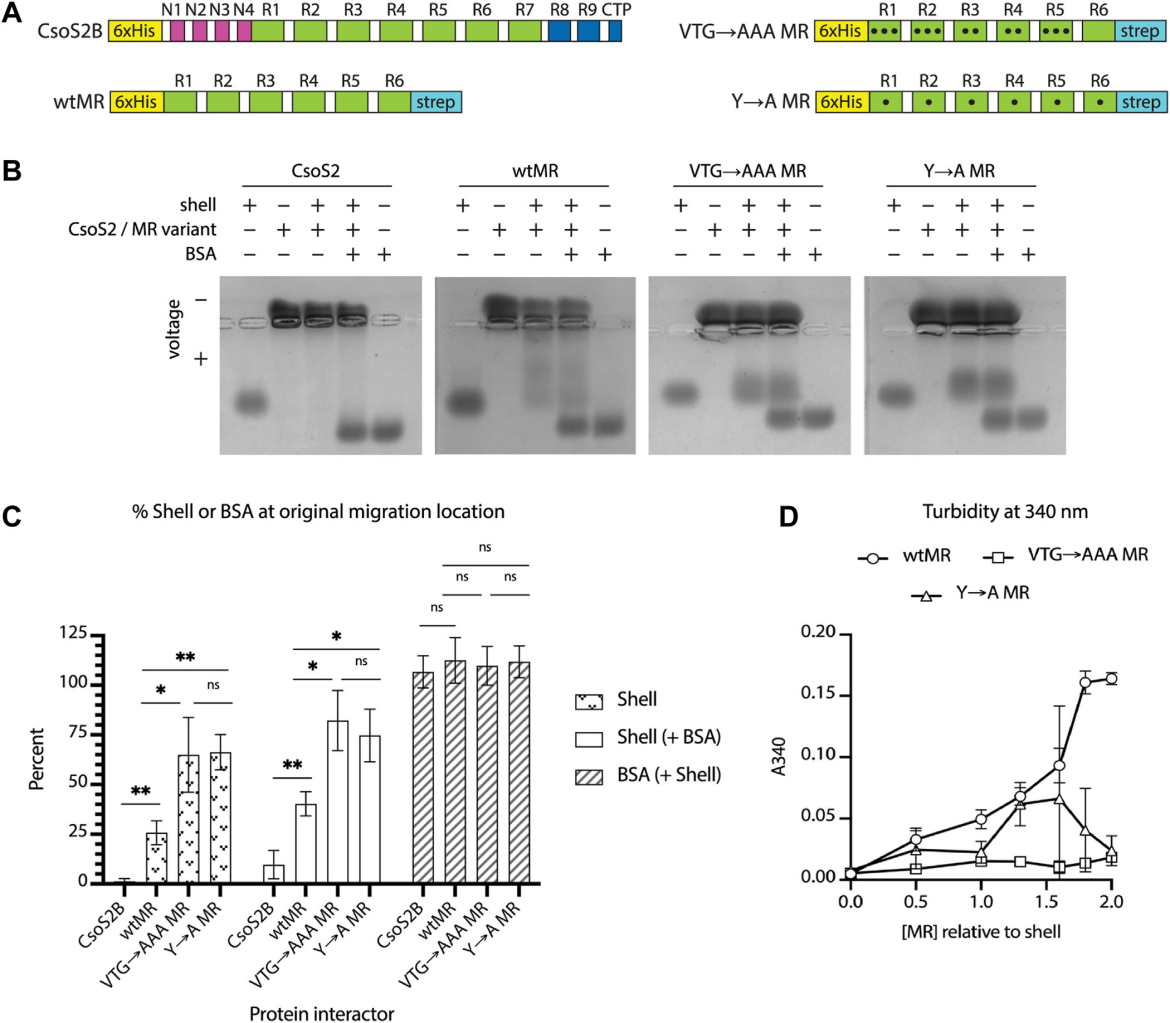
**Shell protein CsoS1A binds to the MR of CsoS2, and mutations to VTG and Y residues perturb binding.***A*, purified constructs used in assays; all constructs have the ribosomal slip-site in R6 mutated to only produce the long form. *Black circles* indicate mutations within a repeat. Cartoons are not to scale with respect to domain sizes. *B*,representative native agarose protein gels of purified shell (CsoS1A) and CsoS2 variants, with BSA as a negative binding control. All gels were run in triplicate. Protein concentrations are as follows: 2.5 μg of shell (CsoS1A) and BSA, 8.4 μg of CsoS2, and 3.5 μg of each MR variant. 8.4 μg of CsoS2 were added instead of 3.5 μg to keep the molar load consistent between CsoS2 and MR samples (0.09 nmols). *C*, gel quantification of the percent shell or BSA at its original migration location (see Fig. S5 for details). Error bars show the SD of three independent samples. Significance of ∗ is *p* ≤ 0.05 in an unpaired *t* test; ∗∗ is *p* ≤ 0.01 in an unpaired *t* test; ns is *p* > 0.05. *D*, turbidity at 10 min of the indicated constructs with defined molar ratios to shell (CsoS1A). All samples contained 9 μM CsoS1A. Concentrations of MR variants were as follows: 0, 4.5, 9, 12, 14, 16, and 18 μM.

Dramatic loss of growth in VTG and Y mutant strains hinted that these MR residues form interactions that are essential for carboxysome assembly. Previous studies showed that full-length CsoS2 binds to shell protein CsoS1A (16), narrowing down candidate MR interaction partners to either shell proteins and/or CsoS2 itself. To biochemically assess the MR's binding interactions, we purified full-length CsoS2 and WT MR (wtMR) along with VTG and Y mutant variations of wtMR (Fig. 3*A*). In purifying wtMR, we wanted to identify MR interactions specifically since the NTD was already known to bind Rubisco (19) and CTD to shell (21, 22, 23). Repeat 7 was left out of the wtMR construct because it occurs after the ribosomal slip site in Repeat 6, in an effort to eliminate potential confounding variables between the CsoS2A and CsoS2B isoforms.

A native agarose electrophoretic mobility shift assay revealed that both CsoS2 and wtMR bind to the hexameric shell protein CsoS1A and that mutating VTG and Y perturbed this interaction (Fig. 3, *B* and *C*). Shell protein showed a dramatic shift in mobility when mixed with full-length CsoS2, with over 90% of the protein binding to CsoS2 and migrating from its control position. Shell was about 25% less mobile when mixed with wtMR, and over 50% of shell remained at the original migration location when mixed with the VTG or Y mutants, indicating a partial loss of binding. Removing the NTD and CTD domains in the wtMR sample, compared to full length CsoS2, had the largest effect on binding to shell, while mutating VTG or Y residues compared to the wtMR sequence showed a more modest, yet still significant, reduction in binding (Fig. 3*C*). A negative control noninteractor, BSA, showed no migration. We did not evaluate the binding to Rubisco or CsoSCA, since it was previously shown that the MR does not interact with either of these proteins (19, 31).

Seeing the binding differences in this qualitative assay, we sought to further investigate the nature of the MR binding interaction. Interactions formed by disordered and/or repetitive proteins can often be monitored by a change in turbidity, which measures the transition from a soluble protein state to phase-separated condensate states. When more wtMR was added to CsoS1A shell protein, turbidity increased in a concentration-dependent manner, while no turbidity was observed for each protein alone (Figs. 3*D* and S6). The mutant VTG construct had little to no turbidity at any concentration, while the mutant Y construct showed intermediate behavior. Full-length CsoS2 displayed almost 5× more turbidity when mixed with shell compared to wtMR, reconfirming the robust contribution of the CTD to shell binding (Fig. S6) (20). Taken together with Figure 3*C*, these results demonstrate that the VTG and Y residues participate in shell binding to the MR, yet they may contribute to the interaction in distinct ways.

### Formation of phase-separated condensates is dependent on the CsoS2 MR sequence

**Figure 4:**
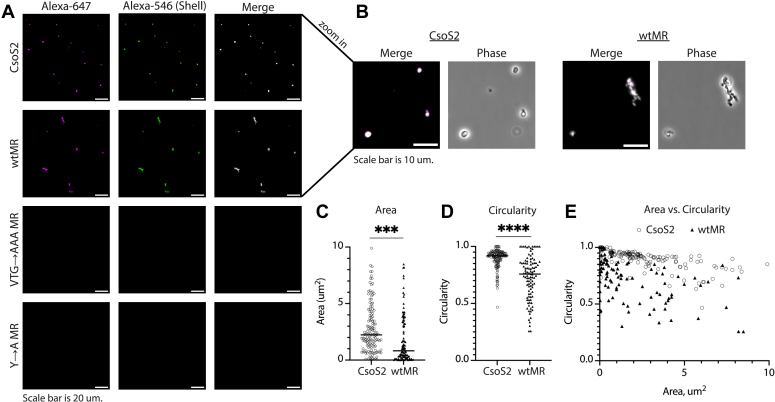
**CsoS2 and wtMR form condensates when mixed with shell but not when key residues are mutated.***A*, fluorescence microscopy of the indicated CsoS2/MR protein variants with added shell (CsoS1A), imaged at 30 min post mixing. All proteins are at a final concentration of 10 μM. All CsoS2/MR variants are labeled in *pink*, shell is labeled in *green*, and the merge appears *white* at equally overlapping intensities. Scale bar represents 20 μm. *B*, zoom-in of CsoS2 and wtMR droplets shown in (*A*). Scale bar represents 10 μm. *C*, comparison of droplet area between CsoS2 and wtMR condensates, measured at 30 min. Significance of ∗∗∗ is *p* ≤ 0.001 in an unpaired *t* test. Each individual droplet appears as an icon on the column scatter plot, and the horizontal line marks the median value. *D*, comparison of droplet circularity between CsoS2 and wtMR condensates, measured at 30 min. Circularity is calculated as 4π∗area/perimeter^2^, with 1.0 being a perfect circle and lower values indicating increasing shape elongation. Significance of ∗∗∗∗ is *p* ≤ 0.0001 in an unpaired *t* test. Each individual droplet appears as an icon on the column scatter plot, and the horizontal line marks the median value. *E*, area versus circularity for all measured CsoS2 and wtMR condensates. For (*C* and *D*), the median is indicated by a *black* line.

Following the results of the turbidity assay, we confirmed *via* fluorescence microscopy that purified CsoS2 and wtMR indeed form phase-separated condensates when mixed with CsoS1A shell protein (Fig. 4*A*). However, when MR with mutated VTG or Y residues was mixed with shell, no condensates formed under these buffer conditions. All experiments were performed at 150 mM salt, mimicking typical intracellular conditions, and no condensates appeared when either CsoS2, MR, or shell was observed on its own (Fig. S10). The addition of 2% PEG-6000 to simulate cellular crowding conditions led to modest, yet visible, phase separation of VTG and Y mutants with shell (Fig. S16). This suggests that these residues may be critical for forming low-affinity, highly multivalent interactions that are quite sensitive to cellular conditions such as salinity and local protein concentrations. In the cell, the bulk chemical potential of these residues may work to drive CsoS2 and shell to phase separate during carboxysome biogenesis.

Interestingly, though both CsoS2 and wtMR formed phase-separated condensates when mixed with shell, the condensates displayed distinct properties in their size and shape (Fig. 4*B*). Both condensates showed accumulated growth over 30 min (Figs. S7–S9) but tended toward divergent shapes over the same growth period (Fig. S9). CsoS2 condensates were larger and more circular on average, while wtMR condensates were smaller and formed elongated structures (Fig. 4, *C*–*E*). The presence or absence of the CTD in the CsoS2 and wtMR constructs is a proxy for CsoS2A and CsoS2B, suggesting that these two proteins may contribute differently to the physical properties of the nascent carboxysome. Condensate shape can be an indicator of a liquid-to-solid phase transition, with liquid droplets often appearing more spherical and solid aggregates appearing more deformed or fibrillar (32, 33). Molecular crowding may also promote an early solid phase transition, as the presence of 2% PEG-6000 led to more aggregate-like structures in all samples (Figs. S15 and S16), including the control of CsoS2 on its own (Fig. S14).

### *In vitro* carboxysome reconstitution and condensate properties

**Figure 5:**
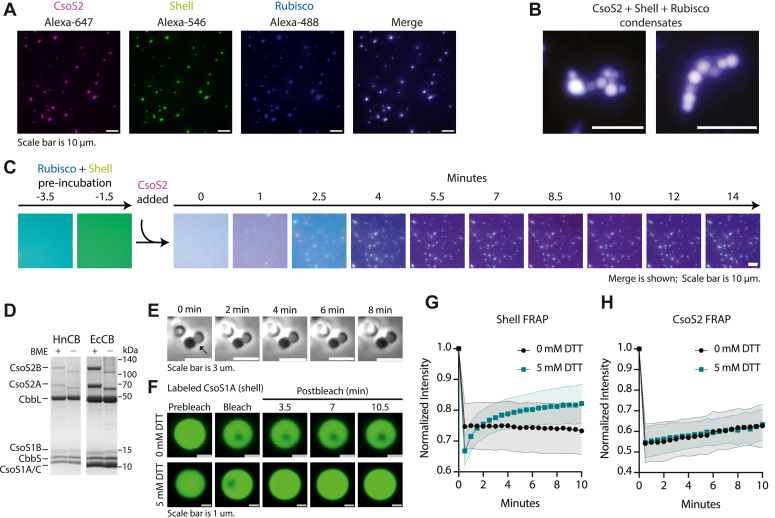
**CsoS2, Shell, and Rubisco form condensates with liquid properties that differ in reducing versus oxidizing conditions.***A*, individual channels of CsoS2 (*pink*) + shell (*green*) + Rubisco (*blue*) condensates, with the merge shown in *white*. Image was taken 30 min post mixing. Final protein concentrations are 7.9 μM Rubisco, 6.1 μM CsoS2, and 17.5 μM shell (CsoS1A). *B*, zoomed-in examples of merged CsoS2 + shell + Rubisco condensates; scale bar represents 10 μm. Zoomed-out images of these condensates are shown in Fig. S12*B*. *C*, time lapse of the addition of CsoS2 (*pink*) to a pre-incubated mixture of Rubisco (*blue*) and Shell (*green*). Rubisco and shell were mixed and observed for 3.5 min, at which point CsoS2 was added and observation continued over 14 min. Images show a merge of three channels, and individual channels from select timepoints can be viewed in Fig. S21. The scale bar represents 10 μm. Final protein concentrations after the addition of CsoS2 are 10 μM Rubisco, 10 μM CsoS2, and 17.5 μM shell. *D*, PAGE gel of carboxysomes (CBs) purified from *Halothiobacillus neapolitanus* (HnCB) and *Escherichia coli* (EcCB) with or without β-mercaptoethanol (BME). CsoS2A and B show distinct downshifts under oxidizing conditions. *E*, phase contrast of CsoS2 + shell + Rubisco condensates that do not merge over 8 min. *F*, example droplets from FRAP showing labeled shell with and without 5 mM DTT. *G*, shell FRAP. *H*, CsoS2 FRAP. *G* and *H*, bleach occurs at ∼30 s; *black* circles, 0 mM DTT; *teal* squares 5 mM DTT; normalized intensity, see Experimental procedures.

Since CsoS2 and shell formed phase-separated condensates *in vitro*, we wanted to see if it was possible to fully reconstitute the carboxysome with its three major constituent components: CsoS2, shell, and Rubisco. The NTD of CsoS2 had been previously shown to form phase-separated condensates with Rubisco at low (20 mM) salt (19) but not yet demonstrated with full-length CsoS2 at physiological salt concentrations (150 mM). CsoS2 and Rubisco formed many small condensates at 5 min post mixing, but these condensates appeared to dissolve back into the soluble phase over 30 min (Figs. S11 and S13). In contrast, when CsoS2, Rubisco, and shell were mixed, they formed robust condensates that grew significantly in size over 30 min (Figs. 5*A*, S12, and S13). Interestingly, with inclusion of the molecular crowding agent PEG, condensate formation between Rubisco and CsoS2 was sustained and even increased over 30 min, though it should be noted that CsoS2 on its own also showed greater phase separation over time with PEG (Fig. S17). With the addition of crowder, the three-component mixture resulted in similarly sized condensates at both early and late timepoints (Fig. S18). This suggests that molecular crowding may be a trigger accelerating CsoS2 and Rubisco interactions, while addition of shell stabilizes condensate growth.

To better understand the 3-component condensate formation, we performed order-of-addition experiments by pre-mixing two components and then adding a third during a time-lapse series in a gasket on a microscope slide (Figs. 5*C* and S19–S21). When noninteracting shell and Rubisco were pre-mixed, no condensates were observed before the addition of CsoS2 (Fig. 5*C*). Condensates appeared within 1 to 2 min after the addition of CsoS2 and settled onto the focused plane of the slide over time, suggesting that droplet growth may occur *via* accretion of individual soluble components (Figs. 5*C* and S21). Similarly, when Rubisco and CsoS2 were pre-mixed, condensates appeared upon the addition of shell (Fig. S19). In contrast, when shell and CsoS2 were pre-mixed, they immediately formed condensates, and added Rubisco did not readily associate with the pre-formed droplets (Fig. S20). Though these order-of-addition experiments do not simulate *in vivo* conditions, in which all proteins are expected to be colocalized in the cell at the same time, they are useful to reveal the shell–CsoS2 interaction as the key driver of phase separation.

Interestingly, condensates were often observed to adhere next to one another without merging over time (Fig. 5, *B* and *E*), a behavior that implied a more gel-like than liquid-like state (32). Protein liquidity in condensates can be sensitive to the solvent chemical environment, including to reducing/oxidizing (redox) conditions (13, 34, 35), which can alter local structure and dynamics *via* changes to reactive moieties like cysteine side chains.

We found that redox-dependent behavior was present in both intact carboxysomes and in reconstitutions. When carboxysomes, purified from either native or heterologous sources, were analyzed by SDS-PAGE under both reducing and oxidizing conditions, CsoS2A and CsoS2B—and no other constituents—display a marked size shift, running as smaller under oxidizing conditions (Fig. 5*D*). Notably, this was a change in size from one homogenous species to another, suggesting that CsoS2 may undergo a specific redox-dependent structural change which could affect interactions between CsoS2 and its binding partners. To test this hypothesis, the liquidity of individual components was assessed using fluorescence recovery after photobleaching (FRAP). FRAP of CsoS2-Rubisco-shell condensates revealed that the shell experiences a dramatic difference in mobility depending on the redox conditions; it had no mobility under oxidizing conditions and recovered under reducing conditions (Fig. 5, *F* and *G*). In contrast, CsoS2 showed nearly identical slow recovery under both oxidizing and reducing conditions (Fig. 5*H*). These results emphasize that the redox environment can independently modulate the mobility of proteins in carboxysome condensates, selectively tuning condensate properties and providing a window into how *in vivo* carboxysome assembly functions.

## Discussion

Carboxysome assembly spans length scales, from single amino acid interactions to thousands of proteins organizing themselves into a >300 MDa compartment. In this work, we dissect each length scale to form a new model of how CsoS2 coordinates assembly of ⍺-carboxysomes. With *in vivo* studies in the native host organism, we identified VTG and Y sequence motifs in the MR as essential for carboxysome assembly. These motifs interact with the shell, an effect which is amplified by high valency across 7 MR repeats. Mutation of key residues *in vivo* and *in vitro* weakened this interaction. We further demonstrate that CsoS2, Rubisco, and shell can be reconstituted *in vitro* into spherical condensates and that the liquidity of the shell can be tuned by the redox environment.

**Figure 6:**
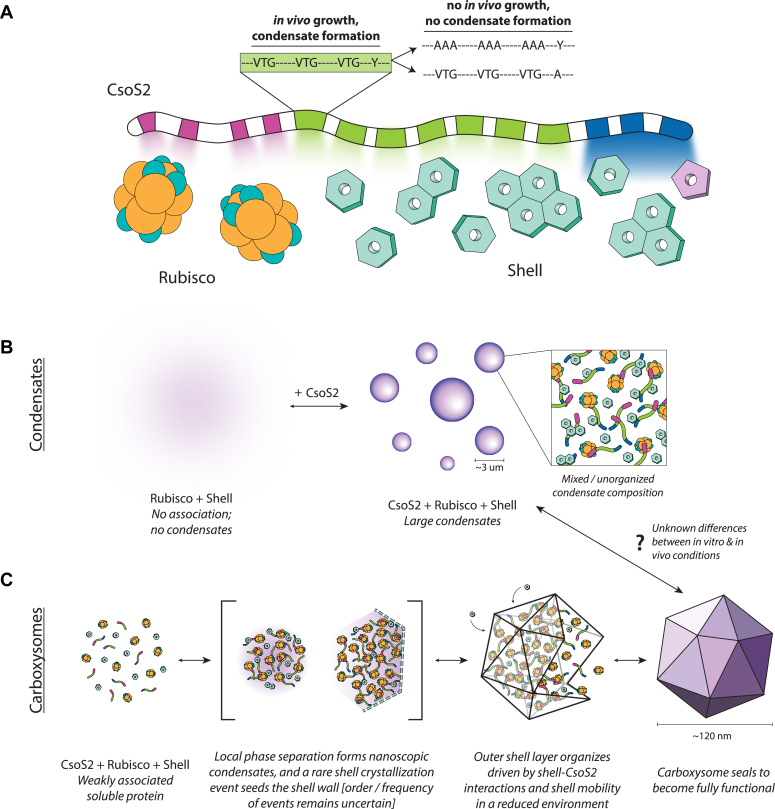
**Model of CsoS2 interactions driving condensate and carboxysome assembly.***A*, cartoon model of known interactions between CsoS2 motifs and binding partners, with colored spotlights highlighting the relative strength of each interaction. *Pink* blocks are NTD repeats, *green* blocks are MR repeats, and *blue* blocks are CTD repeats and the CTP. The pullout box shows the sequence residues that are important for *in vivo* growth and condensate formation and the mutated variants that do not grow or form condensates. *B*, model of condensate formation. Shell and Rubisco have no association at equilibrium but addition of CsoS2 precipitates large condensates. Condensates are assumed to be a mixture of all three proteins with no clear shell layer. *C*, model of *in vivo* carboxysome assembly, informed by condensate biochemistry and previous studies (43, 44). All carboxysome components phase separate locally on a nanoscopic scale, initiating a rare shell crystallization event under as of yet unknown conditions (see text for details). Shell–CsoS2 interactions drive assembly and organization, leading to the formation of sealed and functional compartments. CTP, C-terminal peptide.

Overall, VTG and Y motifs contribute to many weak, transient interactions that en masse increase the binding affinity to shell proteins (Fig. 6*A*). Based on first principles, it is not biochemically obvious how (V/I)(T/S)G motifs facilitate binding to the shell. Mutation to alanine abolished binding, suggesting important contributions from additional alkyl groups as well as the hydrogen bonding interactions from the hydroxyl group. In contrast, there is precedence for the importance of tyrosine residues in IDPs. Tyrosines often participate in pi-pi or cation-pi interactions with other aromatic or charged residues. IDPs with repetitive Y residues such as Fused in Sarcoma (FUS) showed reduced phase separation when greater numbers of Ys were mutated (36, 37, 38), similar to the valency effects we observe for the MR.

The sticker and spacer model has emerged as a useful framework for understanding protein phase separation. In the model, proteins are divided into "sticker" regions responsible for intermolecular interactions and intervening disordered "spacers" (39). The "stickers" may range from single amino acid sidechains (such as the Ys in FUS) all the way to well-defined folded domains such as SSULs in the β-carboxysome scaffold protein CcmM (13). From the recently published structure of the *Prochlorococcus* ⍺-carboxysome, which was able to resolve the CsoS2 MR at ∼4.5 Å local resolution, each MR repeat appears to act as a single binding unit bridging the pseudo-three-fold axis between neighboring hexamers, in which the VTGs form the triangular vertices (23). This structure was also predicted in modeling work from Oltrogge *et al.* (40). In contrast, the CTD binds to shell in a well-conserved yet extended conformation (22, 23).

These recent cryo-EM findings confirm that the MR possesses some structure when bound to shell and that it is distinct from the CTD domain structure. In the structures of the triangular microdomains formed by each MR repeat, the VTGs engage with CsoS1A-His79 in a manner similar to, but distinct from, the extended CTD conformation. The tyrosine positioning in the VTG triads appears to fold away from the MR triangular core and wedge into the three-way junction between hexamers. Though electron density from Zhou *et al.* supports this positioning, the resolution is not sufficient to determine which shell residues the tyrosine may be interacting with. The structures also show that the cysteine residues in each MR repeat are physically adjacent and may form a disulfide bond. This furthers the hypothesis that cysteine oxidation may reinforce and stabilize neighboring shells, corroborating our observations that shell was less mobile in shell-CsoS2-Rubisco condensates under oxidizing conditions (Fig. 5, *F* and *G*) and providing a reason as to why we were unable to purify C→S mutant carboxysomes (Fig. S4).

If MR binds to shell proteins, what is its role in the context of CsoS2B, which includes the additional shell binding CTD domain? Sequence—discussed above—along with valency and charge are three key differences. In *H. neapolitanus*, the MR has a valency of seven repeats, while the CTD only has two. Although these two repeats possess VTGs, they lack the conserved tyrosines, cysteines, and lysines (Fig. S22) and are also followed by the C-terminal peptide. The CTD has a pI of 9.4, making it positively charged at pH values close to seven and promoting interaction with the negative shell luminal interface (20, 22, 23). In contrast, the MR has a pI of 6.2. The roughly 2:1 ratio of MR to CTD (based on the 1:1 ratio of CsoS2A to CsoS2B) additionally amplifies the MR–shell interaction. However, too high of an A:B ratio may be detrimental to carboxysome formation; *in vitro*, MR and shell formed elongated condensates while CsoS2B and shell formed spherical condensates, and *in vivo*, CsoS2A alone is not sufficient to form carboxysomes (24). These differences likely act in concert to give the MR a mode of binding to the shell that is distinct from the CTD. Recent work from Oltrogge *et al.* proposed that the MR repeats bind areas of less shell curvature, that is, the flat shell facets, while the CTD favors higher curvature associated with the vertices. Cryo-EM showing the CTD exclusively bound to pentamer-proximal regions supports this theory (23). The presence of tyrosines and cysteines likely preferences MR repeats to bind to and stabilize three-fold hexamer junctions, while CTD repeats lacking these residues favor a more flexible conformation at the higher curvature junctions nearby to pentamers. In total, the balance of these interactions help set carboxysome size (40).

The ability to study carboxysome assembly both *in vivo* and *in vitro* has many benefits, though it must be acknowledged that carboxysome condensates are not true carboxysomes. They are thousands of times larger in volume, do not contain all carboxysome components at exact ratios found *in vivo*, and lack the architectural organization of an outer shell layer and polyhedral shape. However, they are extremely useful as a proxy tool to biochemically interrogate protein interactions that are challenging to study *in vivo* (Fig. 6*B*). Here we observe that three-component mixtures lead to the most robust condensate formation, identifying the shell–CsoS2 interaction as likely the main driver of local phase separation over weaker CsoS2–Rubisco interactions and the nonexistent Rubisco–shell interaction. Notably, there is no evidence of organization in these condensate assays; we assume CsoS2, Rubisco, and shell are homogeneously mixed.

Extrapolating what this tells us about *in vivo* ⍺-carboxysome formation (Fig. 6*C*), it is known that CsoS2, Rubisco, and shell are transcribed and translated from the same operon in distinct ratios (41, 42). It is thus likely that carboxysome proteins interact immediately during coincident expression. The exact details of carboxysome biogenesis remain uncertain. One pathway posits that these initial interactions drive local phase separation on a nanoscopic scale. Cryoelectron tomography evidence for this is mixed; Rubisco clusters have been observed in *Synechococcus* sp. WH8109 (43) but thus far not in *H. neapolitanus* (44). These same studies both only observe a maximum of one partially assembled carboxysome per cell, which suggests that nucleation of on-pathway assembly is kinetically limited, even if there are multiple phase separation events creating small Rubisco aggregates in the cytosol (Fig. 6*C*, local phase separation panel). Tomographic images of these partially assembled carboxysomes show one to four shell faces with observable packed Rubisco cargo, implying that shell crystallization—that is, the formation of a 2D lattice—nucleates assembly from that site outwards. This further supports a model in which the shell–CsoS2 interaction is the main driver of assembly, rather than a Rubisco-centric model.

In the reduced cytosol, the shell is more mobile and forms interactions with both CsoS2 and itself from within the condensate and *via* outside accretion to organize an outer layer. At a certain volume, it becomes thermodynamically favorable for shell proteins to fully encapsulate the carboxysome condensate, blocking additional growth, and sealing off a functional carboxysome (44, 45). To kinetically trap carboxysome growth or dissolution at a precise size is perhaps even a role of the shell *in vivo*, in addition to concentrating CO_2_. This is the key step where condensates and carboxysomes differ—it is still unknown what branches the completion of a 150 nm compartment from continued growth to a micron-sized particle. It might simply require tweaking of *in vitro* reaction conditions—salt concentration, protein concentration, molecular crowding, *etc.*—to tilt the preference towards smaller compartments. Future work will aim to not only establish the precise conditions to form nm-sized carboxysomes *in vitro* but also to confirm that they can carboxylate CO_2_.

Carboxysomes are a fascinating model system to understand how the coordinated actions of thousands of proteins build an essential cellular structure. Remarkably, the instructions for compartment assembly are encoded solely in the sequences of its constituent proteins. Here we establish that there is a molecular grammar to the CsoS2 MR sequence and how disruption of even a small number of residues diminishes binding to shell and prevents carboxysome formation. This work contributes new motifs to the growing dictionary of known sequence determinants of phase separation and microcompartment formation. Predicting whether a protein will phase separate and form a compartment, along with the conditions that affect this interaction, will continue to be important for informing broader efforts to engineer carboxysomes and other diverse microcompartments in biological systems.

## Experimental procedures

### CsoS2 MR and CTD consensus sequences

All sequences in IMG matching the CsoS2 Pfam (PF12288) were downloaded in May 2020 for a total of 770 sequences. Partial sequences and those with ambiguous residue assignments were discarded, and the set was dereplicated to 95% protein sequence identity using usearch (46). These 272 remaining sequences were analyzed for peptide motifs using the MEME suite (47). The MR and CTD repeat motif positions were identified using MAST for a total of 2190. These repeat sequences were extracted with 15 aa of buffer on either side and then all aligned against each other, including both MR and CTD types, using mafft (48). FastTree was used to build a phylogenetic tree of all the repeats which clearly separated into two major clades: one with MR repeats and one with CTD repeats (49). A number of repeats had been misidentified by MAST as evidenced by their membership in the opposing clade. Notable among these is R7 from *H. neapolitanus* which the phylogeny strongly suggests is actually an MR repeat. The sequences belonging to the MR repeat clade (1662) and CTD repeat clade (528) were aligned again with mafft but this time only against members of their respective clades. Weblogo3 was used to create sequence logos for the two repeat classes from these alignments (50).

### *H. neapolitanus* strain generation

WT *H. neapolitanus* is strain c2, ATCC 23641. The *ΔcsoS2* strain was made by homologous recombination of a spectinomycin resistance cassette into the native CsoS2 locus. Complement and mutant strains were generated by homologous recombination of the new CsoS2 sequence into a neutral site on the genome in the *ΔcsoS2* background strain. The insertion region corresponds to bases 2428660-2429201 on the genome. Plasmids were made by Golden Gate cloning into a neutral site destination vector. The neutral site vector contained the following features from 5′-3': *H. neapolitanus* upstream homology arm (bases 2428121–2428660), KanR, LacIQ, pTRC promoter, gene of interest (CsoS2), rrnB terminator, *H. neapolitanus* downstream homology arm (bases 2429201–2429703). All sequences contained an intact frame-shifting site in CsoS2.

To transform *H. neapolitanus*, 10 ml of DSMZ-68 medium was inoculated per transformation and grown at 30 °C and 5% CO_2_. Cells were collected when the pH indicator had turned gray or light yellow indicating a pH of ∼5.5 to 6.5 (1–2 days of growth). Cells were pelleted at 4000*g* for 10 min at 4 °C and washed with cold milliQ water twice. After the third spin, cells were resuspended in 50 to 100 μl cold milliQ water. Cells were mixed with 500 ng of linearized plasmid and placed in cold electroporation cuvettes, then electroporated at 19 kV/cm, 200 mA, and 25 μF before immediate resuspension in 1 ml cold DSMZ-68 without antibiotic. Recovery occurred during overnight incubation at 30 °C and 5% CO_2_ before plating on DSMZ-68 agar plates containing the selection antibiotic. Colonies usually appeared after 3 to 4 days of growth at 30 °C. The genotype was confirmed *via* colony PCR and sequencing.

A list of *H. neapolitanus* strains with genotype, resistance, and induction can be found in Table S1.

### *H. neapolitanus* selection assays

*H. neapolitanus* was inoculated from a colony on a plate into DSMZ-68 medium with the appropriate antibiotic (none for WT, 10 μg/ml spectinomycin for *ΔcsoS2*, and 10 μg/ml spectinomycin + 2 μg/ml kanamycin for complement/mutant strains) and 100 μM IPTG. Colonies were grown 1 to 2 days in 5% CO_2_ until the medium had turned gray, which corresponded to A600 ∼0.1 to 3. Cells were washed with DSMZ-68 without pH indicator added to collect a more accurate A600. All strains were normalized to A600 of 0.1 and a 10× dilution series was generated. Strains were plated onto dry DSMZ-68 plates with the appropriate antibiotic and 100 μM IPTG and grown in either 5% CO_2_ or air at 30 °C.

### Protein expression and purification

All proteins (6xHis-CsoS2B, 6xHis-wtMR-strep, 6xHis-(VTG→AAA MR)-strep, 6xHis-(Y→A MR)-strep, 6xHis-Rubisco, and strep-CsoS1A) were individually cloned into pET-14–based destination vectors with ColE1 origin, T7 promoter, and carbenicillin resistance (See Table S2 for all protein sequences used in this study). All sequences are from *H. neapolitanus* and correspond to UniProt IDs as follows: CbbL (Rubisco large subunit) O85040; CbbS (Rubisco small subunit) P45686; CsoS2 O85041; CsoSCA (carbonic anhydrase) O85042; CsoS4A (pentameric capsomer) O85043; CsoS4B (pentameric capsomer) O85044; CsoS1C (hexameric capsomer): p45688; CsoS1A (hexameric capsomer): p45689; CsoS1B (hexameric capsomer) P45690; CsoS1D (pseudohexameric capsomer) D0KZ73. For the VTG→AAA MR construct, all VTG and VSG sites were mutated to AAA in repeats 1 to 5. For the Y→A MR construct, all Y sites were mutated to A in repeats 1 to 6. Plasmids were transformed into *E. coli* BL21-AI cells. Cells were grown in LB medium at 37 °C with appropriate antibiotic until mid-log phase (A_600_ of 0.3–0.5), at which point 0.2% L-arabinose was added to induce protein expression and the temperature lowered to 18 °C. Cells were grown overnight before pelleting at 5000*g* the next day and freezing at −20 °C.

Frozen cell pellets were thawed and resuspended in lysis buffer (50 mM Tris, 300 mM NaCl, 20 mM imidazole, pH 7.5) with the addition of 1 mM phenylmethanesulfonyl fluoride (PMSF), 0.1 μl/ml benzonase, and 0.1 mg/ml lysozyme. Cells were lysed on an Avestin EmulsiFlex-C3 homogenizer and clarified at 27,000*g* for 45 to 60 min. All subsequent purification steps were performed at room temperature. Supernatant was added to a Ni-Sepharose resin in a gravity column, washed with wash buffer (50 mM Hepes, 300 mM NaCl, 60 mM imidazole, pH 7.5) and eluted with elution buffer (50 mM Hepes, 300 mM NaCl, 300 mM imidazole, pH 7.5). Proteins with a strep tag (His-wtMR-strep, His-(VTG→AAA MR)-strep, His-(Y→A MR)-strep) were further cleaned up on a Strep-Tactin resin on a gravity column. The entire elution was loaded onto the column, washed with wash buffer (50 mM Hepes, 300 mM NaCl), and eluted with elution buffer (50 mM Hepes, 300 mM NaCl, 2.5 mM d-desthiobiotin) before adding 10% glycerol, flash freezing in liquid N_2_, and storing at −80 °C.

CsoS2B was purified the same way through the His elution step, then further cleaned up using size-exclusion chromatography. Eluted protein was loaded onto a HiPrep 16/60 Sephacryl S-200 HR column equilibrated in 50 mM Hepes, 300 mM NaCl buffer on an Akta Pure chromatography system. Fractions with full-length protein were concentrated on Amicon Ultra 15 Ultracel 30K filters before adding 10% glycerol, flash freezing in liquid N_2_, and storing at −80 °C.

Strep-CsoS1A was lysed and clarified the same way as above in 50 mM Hepes, 150 mM NaCl lysis buffer, then purified on a Strep-Tactin resin on a gravity column. Clarified lysate was loaded onto the column and allowed to flow through, followed by a wash step and elution with elution buffer (50 mM Hepes, 150 mM NaCl, 2.5 mM D-desthiobiotin) before adding 10% glycerol, flash freezing in liquid N_2_, and storing at −80 °C.

6xHis-Rubisco was lysed and clarified the same way as above yet with a different lysis buffer (50 mM Tris, 150 mM NaCl, 20 mM imidazole, pH 7.5) and purified on a Ni-Sepharose resin on a gravity column the same way as above. Wash buffer was 50 mM Tris, 150 mM NaCl, 60 mM imidazole, pH 7.5. Elution buffer was 50 mM Tris, 150 mM NaCl, 300 mM imidazole, pH 7.5. Eluted protein was buffer exchanged on a 2 ml Zeba desalting column before adding 10% glycerol, flash freezing in liquid N_2_, and storing at −80 °C.

### Carboxysome expression and purification

Carboxysomes were expressed in *E. coli* BW25113 off of the pHnCB10 plasmid (as described in Bonacci *et al.* (9)) with 500 μM IPTG induction at mid-log phase. Cells were grown overnight at 18 °C and pelleted the next day. Carboxysomes were purified as described previously (19). Briefly, cell pellets were lysed using B-PER reagent with the addition of 1 mM PMSF, 0.1 μl/ml benzonase, and 0.1 mg/ml lysozyme. Lysis took place for 45 min at room temperature while shaking. Lysate was spun for 20 min at 12,000*g*, the supernatant collected, and then spun again for 30 min at 40,000*g* and the supernatant discarded. The pellet was resuspended in 200 μl TEMB (10 mM Tris, 10 mM MgCl2, 1 mM EDTA, pH 8.0) on ice with gentle rocking for 1 h to overnight, with additional resuspension *via* pipette if needed. The resuspended pellet was clarified for 3 min at 1000*g* before loading onto a 5-step sucrose gradient (10, 20, 30, 40, and 50% w/v sucrose in TEMB). Gradients were spun for 15 min at 105,000*g* or longer depending on the size of the prep. The gradient was fractionated and analyzed with SDS-PAGE. Fractions containing carboxysomes were pooled and centrifuged for 30 to 90 min at 105,000*g*, resuspended in TEMB, and stored at 4 °C. The SDS-PAGE gel in Figure 5*C* was run with 1% (v/v) β-mercaptoethanol.

### Native agarose protein gels

Gels were made from Tris-acetate-EDTA buffer with 1% agarose. Protein samples were mixed (buffer: 50 mM Hepes, 150 mM NaCl) and allowed to equilibrate to room temperature over 30 min before adding native loading dye. Samples were not boiled. Protein concentrations were as follows: 2.5 μg of CsoS1A (shell), 8.4 μg of CsoS2, 3.5 μg of wtMR, 3.5 μg of VTG→AAA MR, 3.5 μg of Y→A MR, and 2.5 μg of BSA. 8.4 μg of CsoS2 were added instead of 3.5 μg to keep the nmol load consistent between CsoS2 and MR samples (0.09 nmols). Gels were run for 70 min at 60 V in native buffer (25 mM Tris, 200 mM Glycine, pH 7.5). Gels were stained for 1 h with Gel Code Blue, then destained with water until most of the stain had dissipated from the background. Gel quantification was done in FIJI.

### Turbidity assays

Protein was thawed to room temperature before mixing. All samples were prepared to a final buffer composition of 50 mM Hepes, 150 mM NaCl. All samples contained 9 μM CsoS1A (except for the 0 μM shell sample). Concentrations of MR variants were as follows: 0, 4.5, 9, 12, 14, 16, and 18 μM. The 0 μM shell control had 18 μM of MR. For CsoS2, concentrations tested were 4.5 and 14 μM. The 0 μM shell control had 14 μM of CsoS2. Forty microliters were pipetted into a Nunc 384 well transparent plate and data collected on a Tecan Spark plate reader.

### Condensate microscopy and quantification

Strep-CsoS1A (shell) was labeled with Alexa546 NHS Ester, at a ratio of 2× dye to hexamer. All CsoS2 and MR variants were labeled with Alexa647 NHS Ester, at a ratio of ⅙ dye to monomer. Rubisco was labeled with Alexa488 TFP Ester at a ratio of one-third dye to L8S8 hexadecamer. Prior to dyeing, Rubisco was buffer exchanged into 50 mM Hepes, 150 mM NaCl buffer on a Spin-X UF Corning 100K 0.5 ml filter tube. Labeling occurred for 1 h in the dark at room temperature. Thermo Fluorescent Dye Removal Columns (#22858) were used to wash away the unconjugated dye, using an equal amount of resin to the volume of the sample. Proteins were thawed to room temperature before mixing in a PCR tube in a final buffer concentration of 50 mM Hepes, 150 mM NaCl. All proteins were at a concentration of 10 μM, except for the Rubisco+CsoS2+shell sample, which had 7.9 μM Rubisco, 6.1 μM CsoS2, and 17.5 μM shell. For crowding experiments, PEG-6000 was used at a 2% final concentration. At 5 min and 30 min, a 1.3 μl sample was taken from the tube and pipetted onto a microscope slide (VWR micro cover glass 24 x 60 mm No.1) and a coverslip added (VWR micro cover glass 24 x 30 mm No.1). For the gasket addition experiments (Figs. S19–S21), 1 to 2 μl of protein was added to 10 to 11 ul of pre-incubated protein (for a total of 12 ul) at the indicated timepoint and subsequently imaged. For Fig. S19 (Rubisco + S2 add shell), final concentrations were 8.3 μM Rubisco, 8.3 μM CsoS2, and 17.5 μM shell. For Fig. S20 (CsoS2 + shell add Rubisco), final concentrations were 10 μM Rubisco, 10 μM CsoS2, and 17.5 μM shell. For Figs. 5*C* and S21 (Rubisco + shell add S2), final concentrations were 10 μM Rubisco, 10 μM CsoS2, and 17.5 μM shell. Samples were imaged on a Zeiss Axio Observer Z1 inverted fluorescence microscope at 100× magnification with an oil immersion objective. The gasket in Figs. S19–S21 is a Coverwell Perfusion Chamber 8x9mm diameter by 0.9 mm depth (#622105). The Alexa546 channel appears as *green*, the Alexa647 channel appears as *magenta*, and the Alexa488 channel appears as *blue*. All images were analyzed in FIJI. The intermodes thresholding algorithm was used to define droplets and make a mask before taking measurements. Condensates under 0.002 um^2^ and over 10 um^2^ were discarded due to false positives of misclassified droplets during the thresholding process.

### FRAP measurements

FRAP experiments were done on a Leica STELLARIS 5 microscope with a white light laser. Each image was taken as a z-stack. A pre-bleach image was taken, and then droplets were bleached at 499, 557, and 653 nm at 40% laser intensity. A post-bleach timelapse took an image every 30 s for 10 min. For image analysis, images were first converted into average projections using the LASX microscope software, then further analyzed on FIJI. Drift correction was applied to each channel using StackReg (translation), then the background subtracted using a 50 pixel radius. FRAP measurements were analyzed using the method described in Guillén-Boixet *et al.* (51).

### Western blots

Five milliliters of each strain were grown at 30 °C and 5% CO_2_ (except for WT which was grown in air) in DSMZ-68 medium with appropriate antibiotic and with or without 100 μM IPTG. At early log phase (indicated by gray or light yellow pH indicator in the medium), cells were pelleted at 4000*g* for 10 min, the supernatant discarded, and frozen at −20 °C for later analysis. For analysis, cells were thawed with 200 ml B-PER reagent, plus 1 mM PMSF, 0.1 μl/ml benzonase, and 0.1 mg/ml lysozyme (final concentrations). Lysis occurred over 45 min at room temperature while shaking. Samples were mixed with loading dye containing β-mercaptoethanol and boiled for 6 min. For the blot in (a), ∼25 μg of protein was loaded per well (± 3 μg), for (b) 25 μg, and for (c) 50 μg (12.5 μg for WT). Samples were run on a Biorad TGX 4 to 20% gel for 40 min at 180 V. The gel was rinsed in water before transferring onto a PVDF membrane using a Biorad TransBlot Turbo for 10 min at 2.5 A and 25 V. The membrane was blocked in TBST (50 mM Tris pH 7.5, 150 mM NaCl, 0.1% Tween-20, 5% rehydrated milk) overnight at 4 °C while shaking. The next morning, the buffer was replaced with 10 ml new TBST (2.5% milk) and primary antibody added (polyclonal rabbit antibodies ordered from GenScript). The antibody used for each blot is indicated in the figure. Both antibodies were added at a 1:2000 dilution. Blots were incubated with primary for 1 h at room temperature, rinsed 3× with TBST, then incubated with secondary (Goat-HRP anti-Rabbit IgG) at 1:10,000 dilution in TBST (1% milk) for 1 h. Blots were rinsed 3× with TBST for 15 min before adding 12 ml of BioRad Clarity Western ECL Substrate, incubating for 5 min, and imaging.

## Data availability

All data is contained within the manuscript. Data is available to share upon request. Please contact the corresponding author.

## Supporting information

This article contains supporting information.

## Conflict of interest

D. F. S. is a co-founder and scientific advisory board member of Scribe Therapeutics. All other authors declare that thye have no conflicts of interests with the contents of this article.
