# Supplementary material for: Conserved and repetitive motifs in an intrinsically disordered protein drive ⍺-carboxysome assembly[image]
Source: J Biol Chem. 2024 Jul 4;300(8):107532. doi: 10.1016/j.jbc.2024.107532 (PMC11365436; doi:10.1016/j.jbc.2024.107532)
Supplement: Supplemental Figures and Tables legends [file mmc1.pdf]

Conserved and repetitive motifs in an intrinsically disordered protein drive α-carboxysome assembly

Julia B. Turnšek, Luke M. Oltrogge, David F. Savage

Figures:

Page	Figure	Description
1	S1	CsoS2 knockouts in *H. neapolitanus* are partially complemented in air.
2	S2	Illustration of all CsoS2 variants expressed *in vivo* in *H. neapolitanus*.
3	S3	Western blots of complemented CsoS2 and mutant strains.
4	S4	Carboxysomes with C→S mutations cannot be purified from *E. coli*.
5	S5	Quantification of native agarose gels.
6	S6	Shell protein CsoS1A binds CsoS2 with high affinity.
7	S7	Smaller condensates at 5 minutes after mixing.
8	S8	Larger condensates at 30 minutes after mixing (replicate set).
9	S9	Over 30 minutes condensate area increases for both CsoS2 and wtMR, but condensates develop into different shapes.
10	S10	Individual protein controls show no condensate formation.
11	S11	Rubisco and CsoS2 form condensates that dissociate over time.
12	S12	Rubisco, CsoS2, and CsoS1A form robust spherical condensates that grow in size over 30 minutes.
13	S13	Addition of CsoS1A leads to an increase in droplet size.
14	S14	2% PEG leads to condensate formation in some controls.
15	S15	2% PEG leads to some condensate formation of CsoS2 and MR variants with shell at 5 minutes post-mixing.
16	S16	2% PEG leads to some condensate formation of CsoS2 and MR variants with shell at 30 minutes post-mixing
17	S17	2% PEG leads to CsoS2 and Rubisco condensate formation at 5 and 30 minutes post-mixing.
18	S18	2% PEG leads to Rubisco, CsoS2, and CsoS1A condensate formation at 5 and 30 minutes post-mixing.
19	S19	Adding shell to Rubisco + CsoS2 nucleates condensate formation.
20	S20	Shell + CsoS2 nucleates condensate formation, and Rubisco does not appear to add to condensates.
21	S21	Adding CsoS2 to Rubisco + Shell nucleates condensate formation.
22	S22	CTD repeat consensus motif.

Tables:

23	S1	Strains used in *H. neapolitanus* growth experiments.
24	S2	Sequences used in this study.

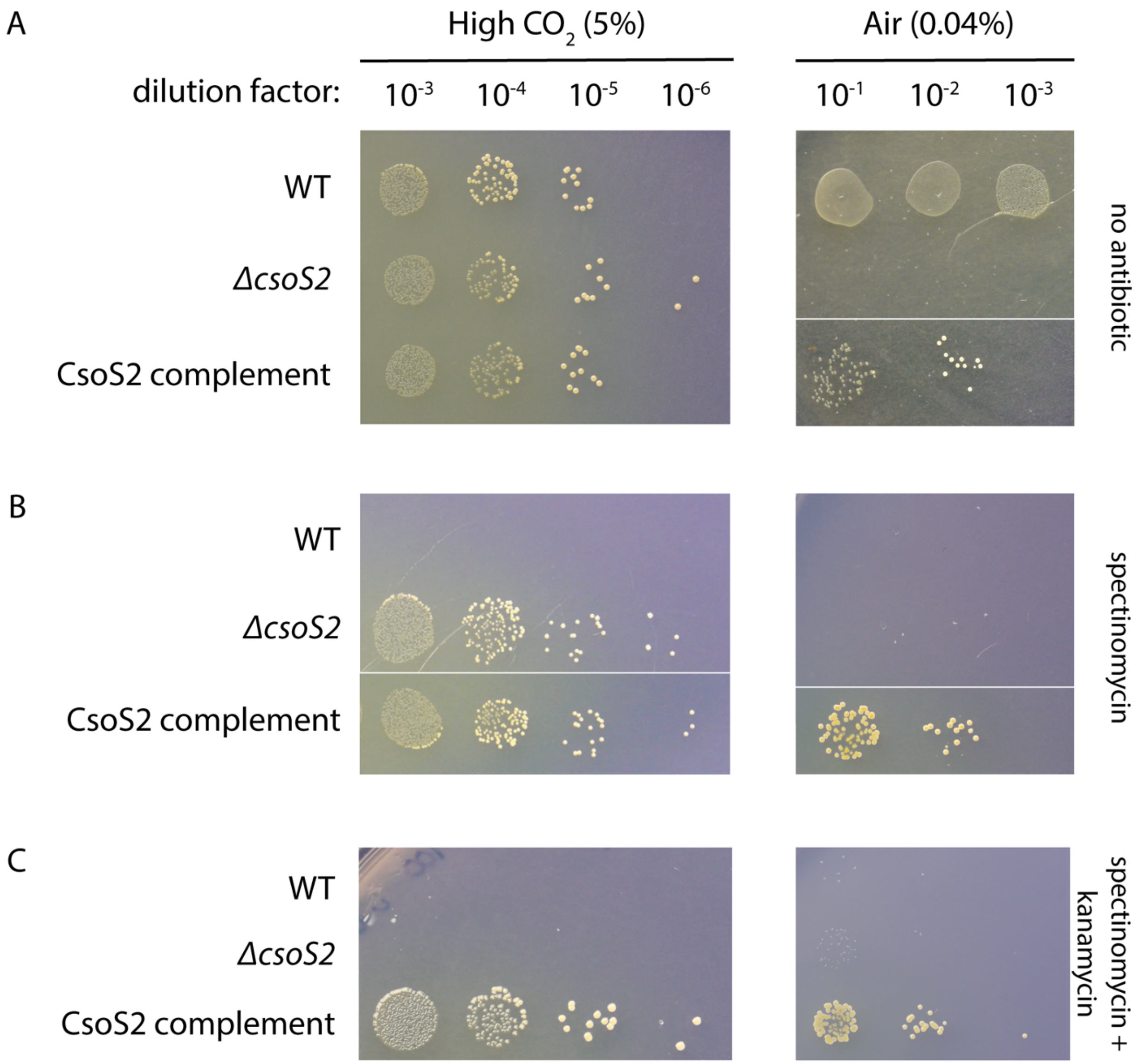

Figure S1. CsoS2 knockouts in *H. neapolitanus* are partially complemented in air. The *ΔcsoS2* strain is a knockout of CsoS2 via spectinomycin ORF insertion. The CsoS2 complement strain contains a genomic insertion of IPTG-inducible CsoS2 and kanamycin targeted to a neutral site on the genome in the *ΔcsoS2* background strain. WT, *ΔcsoS2*, and CsoS2 complement strains were plated in a serial dilution and grown in high CO_2 (5% CO_2) or air (0.04% CO_2) on DSMZ68-agar plates + 100 µM IPTG with (A) no antibiotic, (B) 10 µg/ml spectinomycin, and (C) 10 µg/ml spectinomycin + 2 µg/ml kanamycin. Some images are a composite from different rows on the same plate that were not immediately next to each other, as marked by a thin white line.

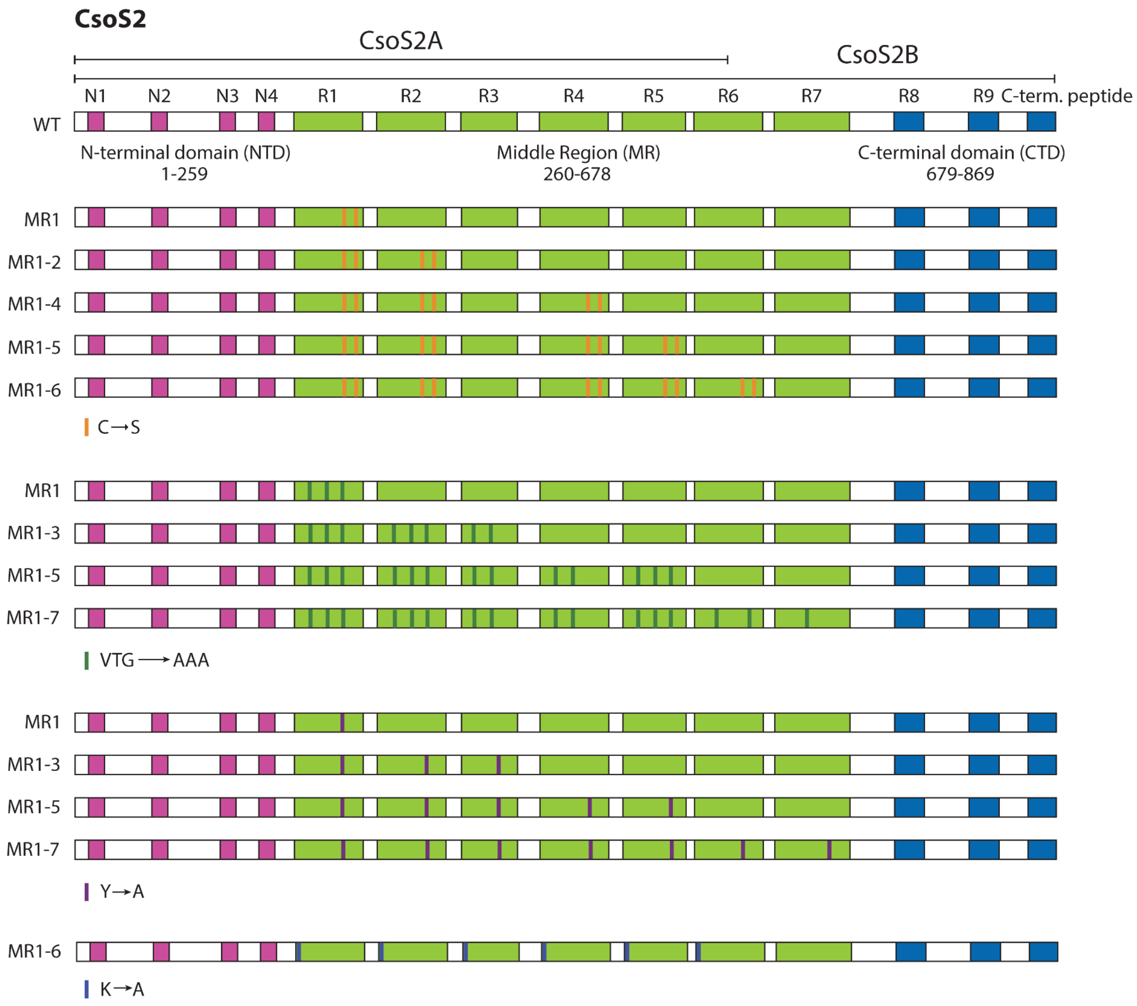

Figure S2. Illustration of all CsoS2 variants expressed *in vivo* in *H. neapolitanus*. Note for the C→S variants, there are no cysteines in R3. Note for the VTG variants, only VTG and VSG sites were mutated.

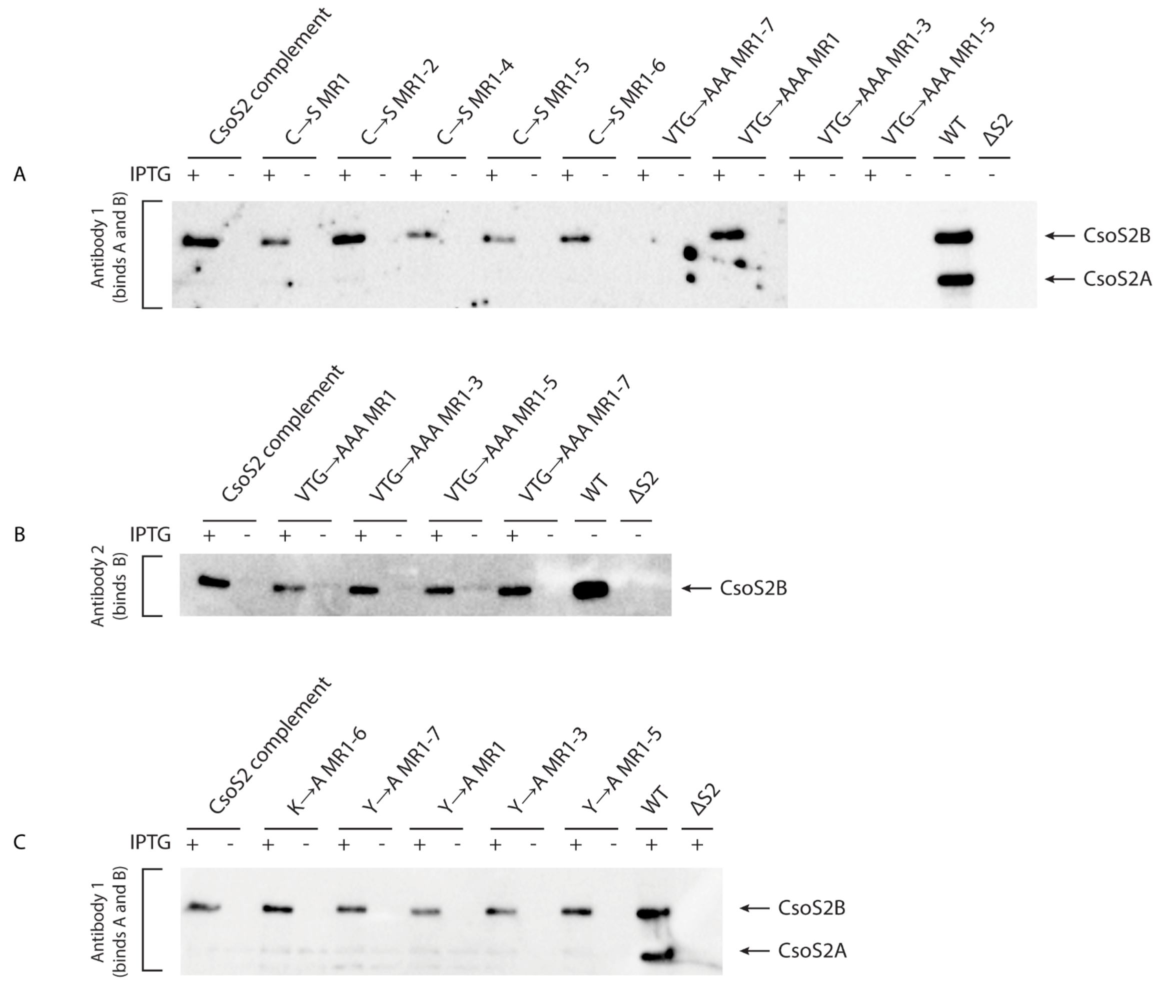

D

Anti-CsoS2 antibodies	Peptide epitope	Reacts with
Antibody 1	CGTEVSRTEKVTGNE	CsoS2A and CsoS2B
Antibody 2	CANRNVPKPEKPGSK	CsoS2B

Figure S3. Western blots of complemented CsoS2 and mutant strains. Strains were grown in liquid culture at high CO_2 (5%) +/- 100 µM IPTG and appropriate antibiotics. (A) C→S and VTG→AAA mutants blotted with antibody 1; loading was normalized to ~25 µg/sample. Some antibodies do not bind due to the mutated VTG sequence in the strain. (B) VTG→AAA mutants blotted with antibody 2; loading was normalized to 50 µg/sample. WT was loaded at 12.5 µg to reduce underexposure. (C) K→A and Y→A mutants blotted with antibody 1; loading was normalized to 25 µg/sample. (D) Table of antibodies and their binding epitopes with VTG underlined.

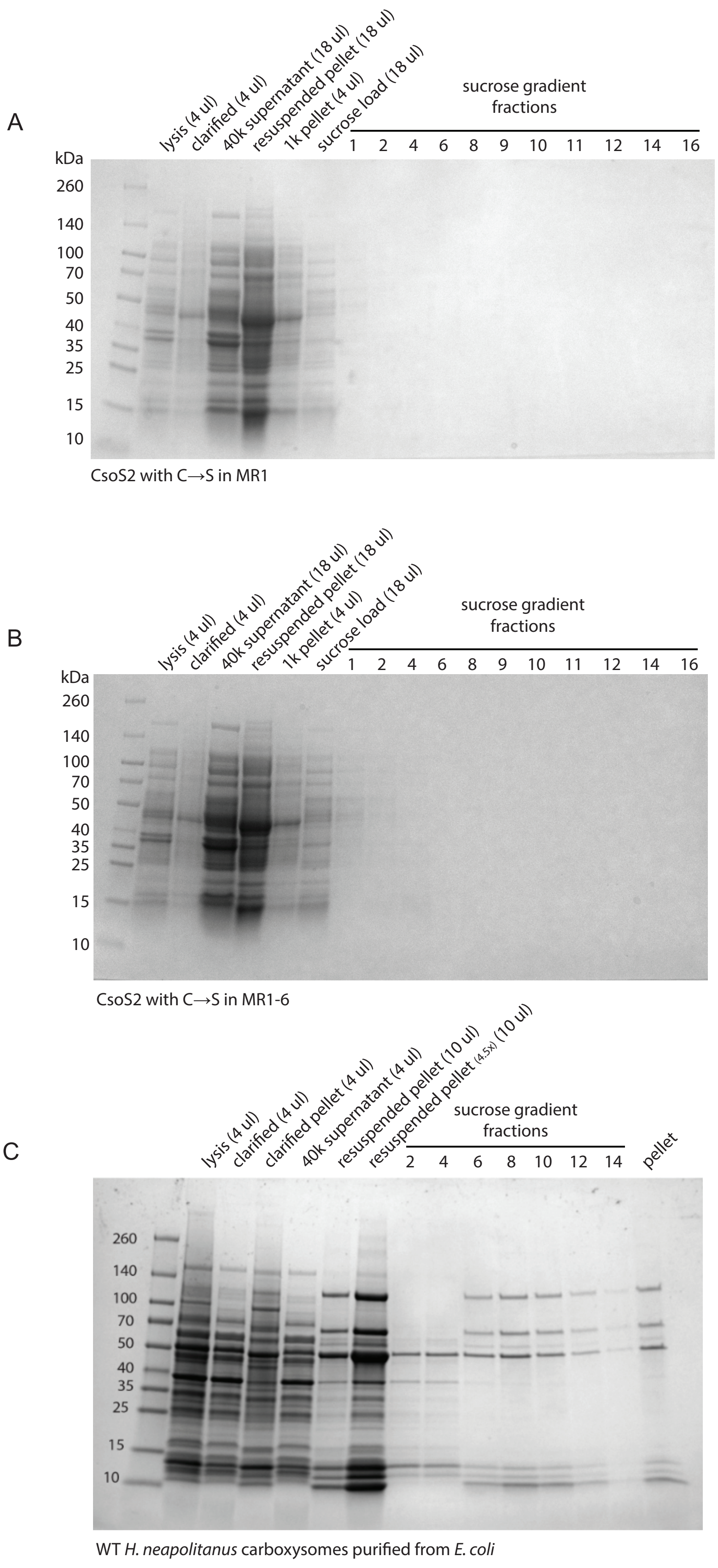

Figure S4. Carboxysomes with C→S mutations cannot be purified from *E. coli*. The indicated CsoS2 C→S mutants were expressed in *E. coli* from a plasmid containing the *H. neapolitanus* carboxysome operon and purified using standard methods (see Experimental Procedures for details). PAGE gel of the carboxysome purification with (A) the CsoS2 variant C→S in MR1, (B) the CsoS2 variant C→S in MR1-6, and (C) wild-type carboxysomes. Carboxysome bands would be expected to appear around fractions 6-12 in (A) and (B). Both CsoS2 mutant preps were attempted a second time with similar results.

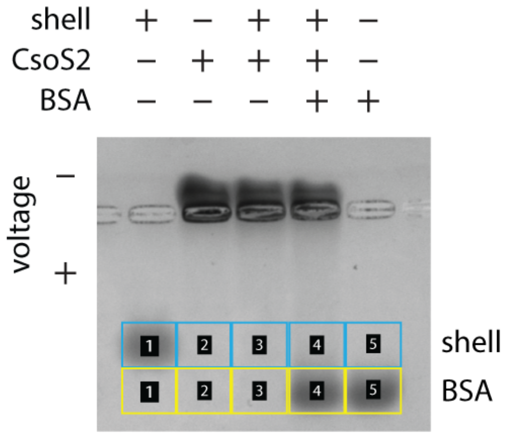

Figure S5. Quantification of native agarose gels. Representative gel (same CsoS2 agarose gel that is in Figure 3B), with boxes drawn around quantified areas. Blue boxes indicate areas of the gel that were quantified to measure shell migration, and yellow boxes indicate areas of the gel that were quantified to measure BSA migration. For shell, density in boxes 2 and 5 were averaged and subtracted as background, since there was no shell present in these samples. For BSA, density in boxes 1, 2, and 3 were averaged and subtracted as background, since no BSA was present in these samples. Three gels for each sample were run and quantified in this way, which is summarized in Figure 3C.

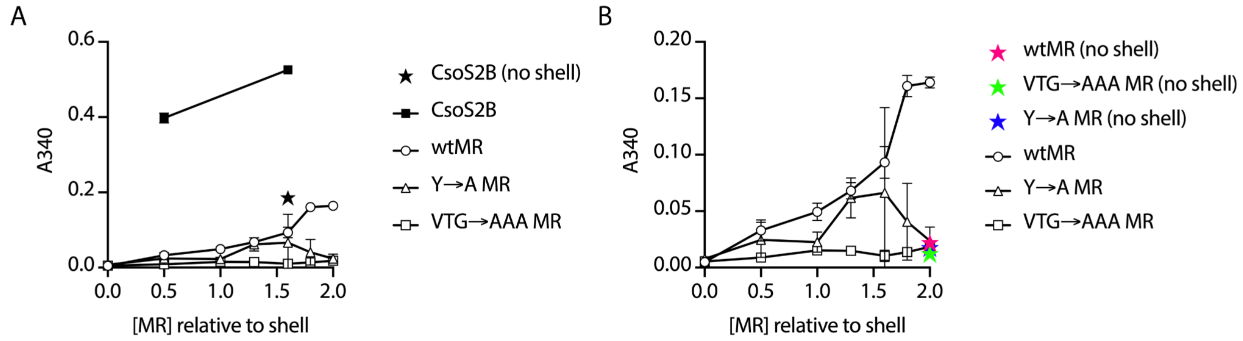

Figure S6. Shell protein CsoS1A binds CsoS2 with high affinity. Turbidity assay at 10 minutes of the indicated constructs with CsoS1A. Data are the same as in Figure 3C, but with CsoS2B shown for comparison (A) and no shell controls (A and B). Unobservable error bars are smaller than the datapoint icon. All samples contained 9 µM CsoS1A (except for the 0 µM shell sample). Concentrations of MR variants were: 0, 4.5, 9, 12, 14, 16, and 18 µM. The 0 µM shell control had 18 µM of MR. For CsoS2, concentrations tested were 4.5 and 14 µM. The 0 µM shell control had 14 µM of CsoS2.

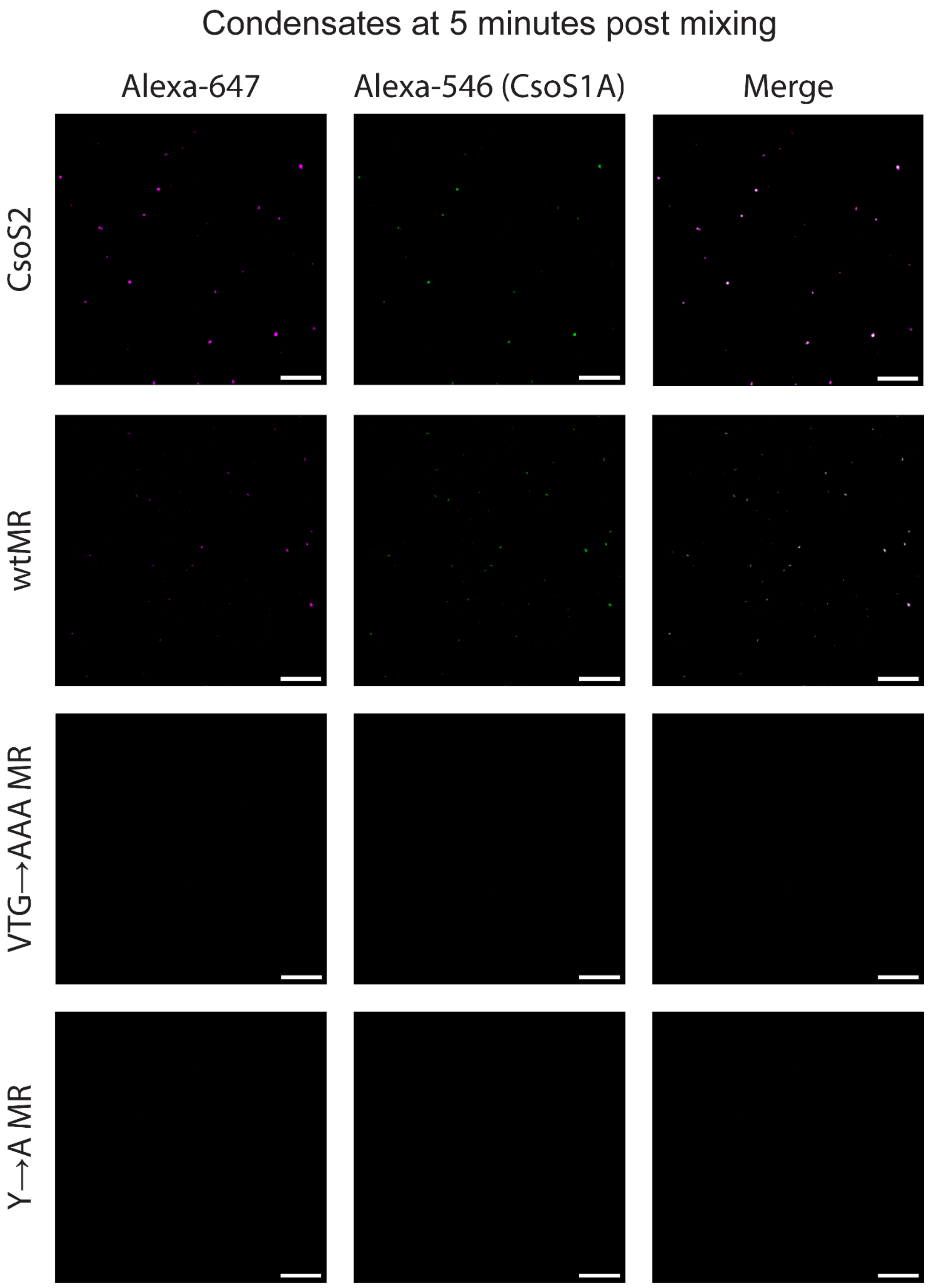

Figure S7. Smaller condensates at 5 minutes after mixing. Fluorescence microscopy of the indicated CsoS2 / MR protein variants with added shell (CsoS1A), imaged at 5 minutes post mixing. All CsoS2 / MR variants are labeled in pink, shell is labeled in green, and the merge appears white at equally overlapping intensities. All proteins are at a final concentration of 10 µM. Scale bar is 20 µm.

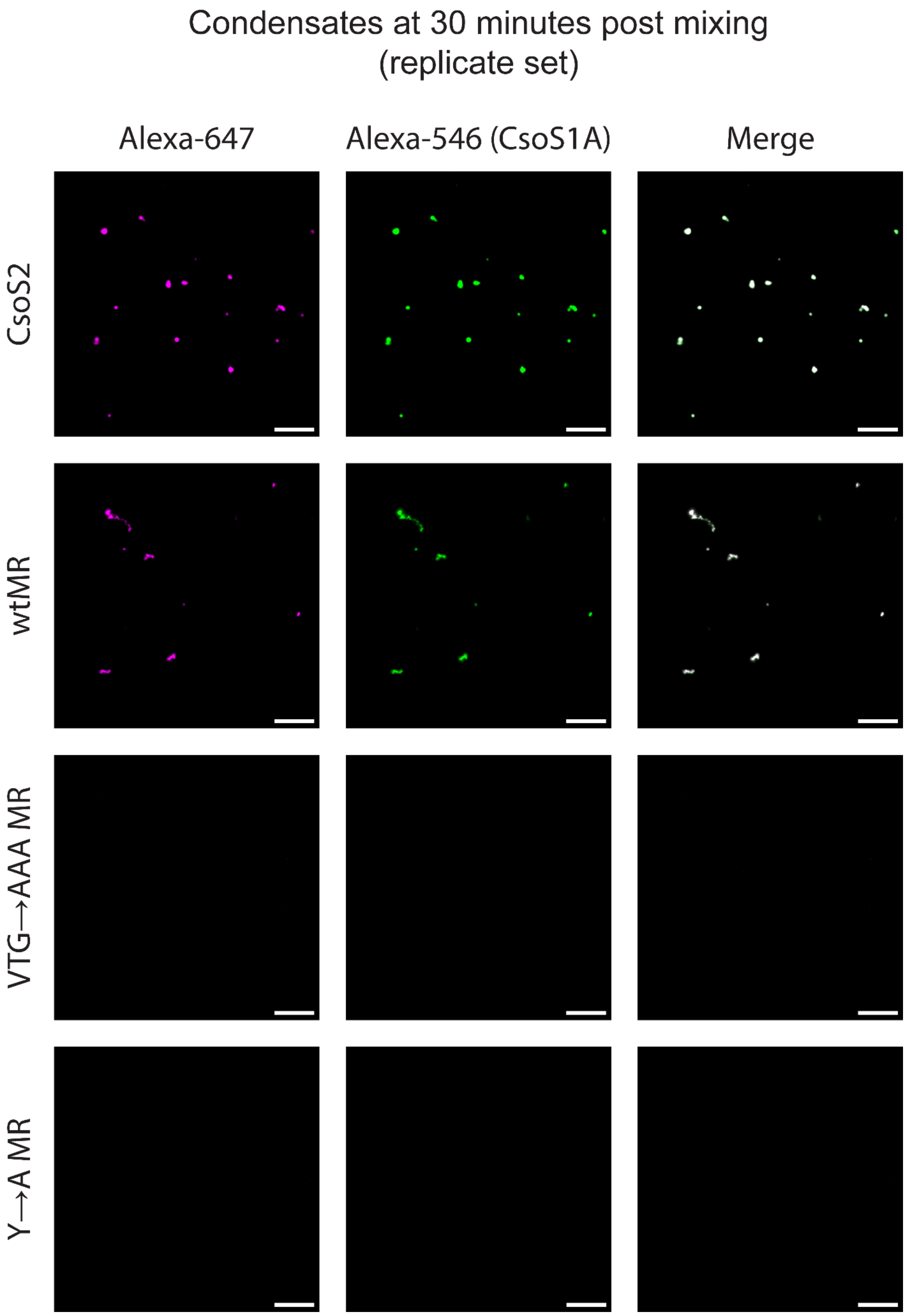

Figure S8. Larger condensates at 30 minutes after mixing (replicate set). Replicate set of micrographs in Figure 4 of the indicated CsoS2 / MR protein variants with added shell (CsoS1A), imaged at 30 minutes post mixing. All CsoS2 / MR variants are labeled in pink, shell is labeled in green, and the merge appears white at equally overlapping intensities. All proteins are at a final concentration of 10 µM. Scale bar is 20 µm.

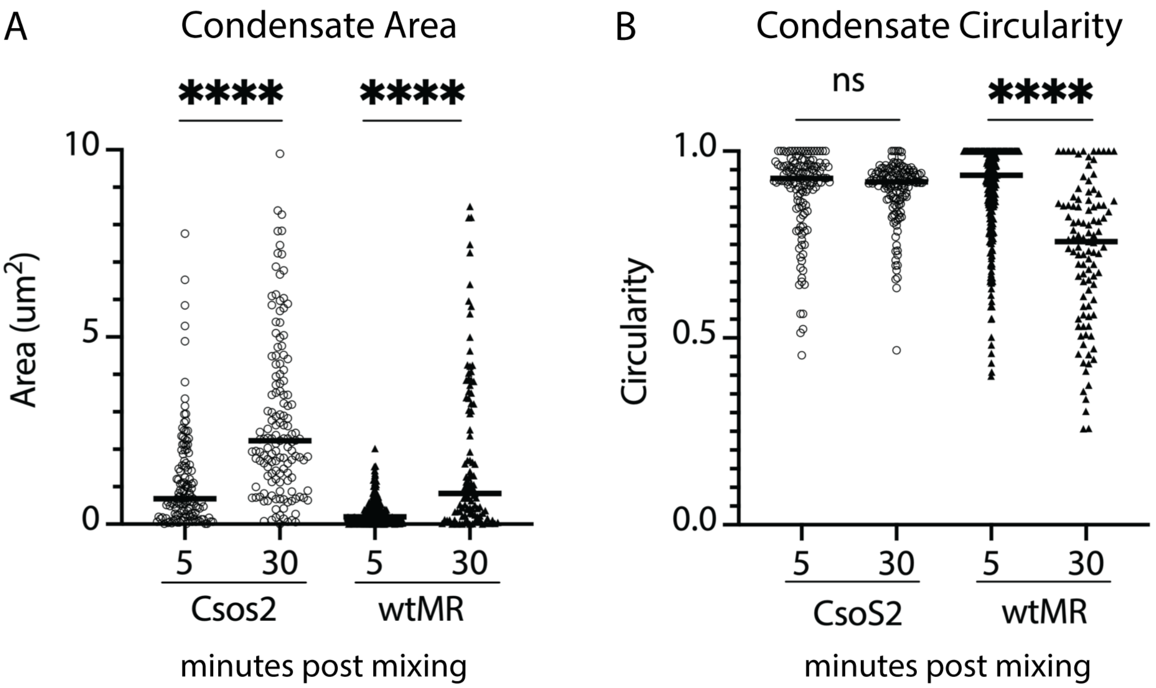

Figure S9. Over 30 minutes condensate area increases for both CsoS2 and wtMR, but condensates develop into different shapes. (A) Condensate area in μm^2 at 5 minutes and 30 minutes post mixing with shell (CsoS1A) for both CsoS2 and wtMR. (B) Condensate circularity at 5 minutes and 30 minutes post mixing with shell for both CsoS2 and wtMR. Circularity is calculated as 4π*area/perimeter^2, with 1.0 being a perfect circle and lower values indicating increasing shape elongation. The median is indicated by a black line. Each individual droplet appears as a dot on the plot. Significance of **** is $P \leq 0.0001$ in an unpaired t-test.

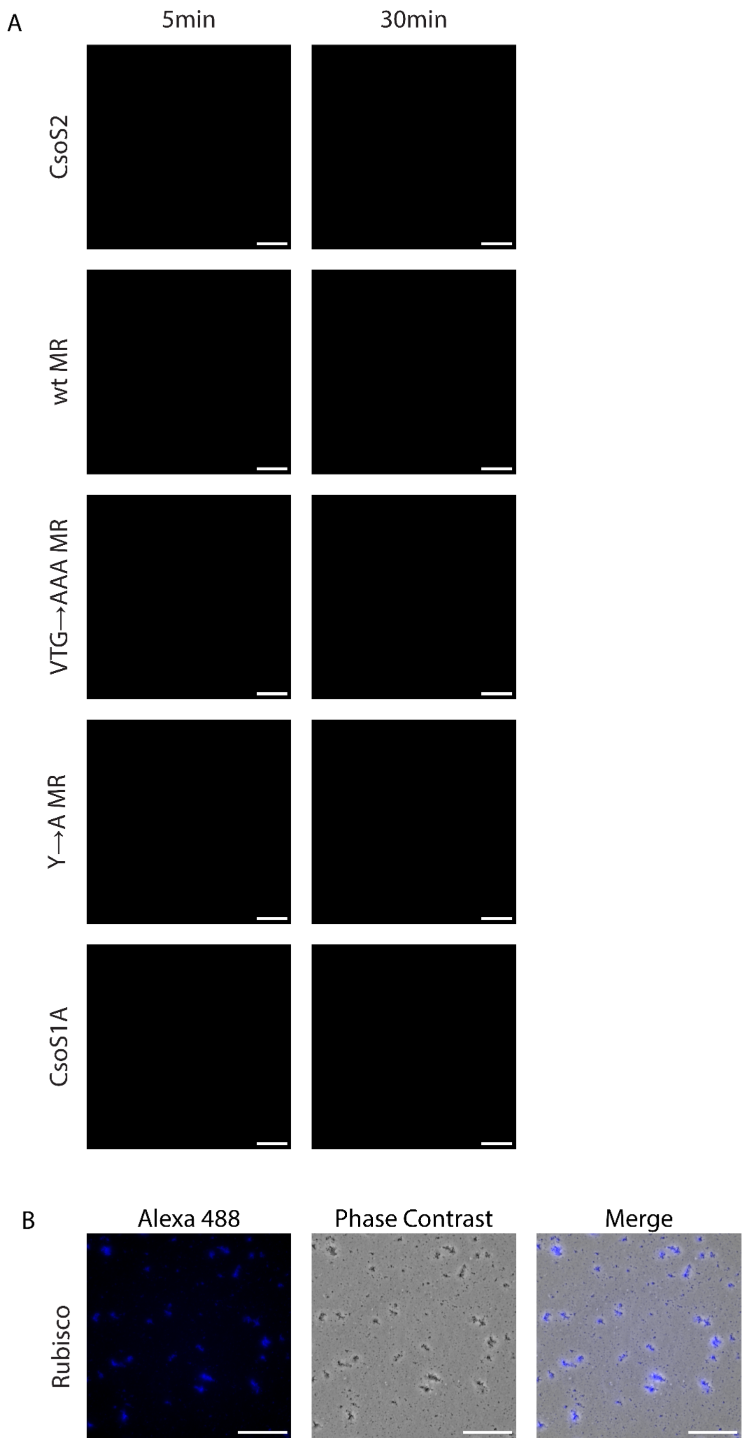

Figure S10. Individual protein controls show no condensate formation. (A) Each indicated protein construct was imaged at 5 minutes and 30 minutes. CsoS2 and MR variants were imaged on the Alexa-647 channel. CsoS1A was imaged on the Alexa-546 channel. All proteins are at a final concentration of 10 µM. All scale bars are 20 µm. (B) Rubisco imaged at 5 minutes on the Alexa-488 channel and phase contrast, and an image merge. Rubisco concentration is 10 µM. Scale bar is 20 µm.

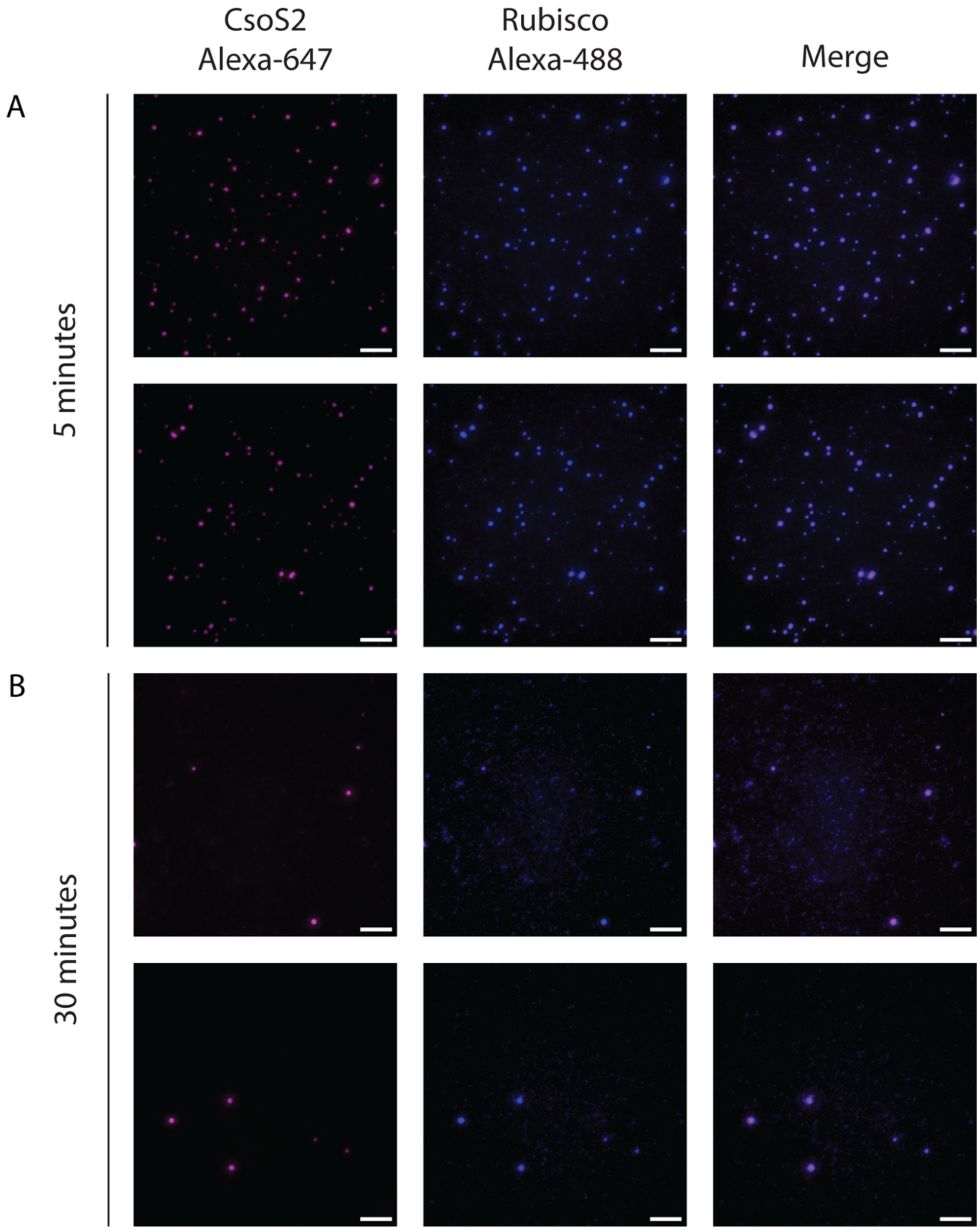

Figure S11. Rubisco and CsoS2 form condensates that dissociate over time. CsoS2 is labeled in pink, Rubisco in blue, and the merge is shown in purple. (A) 5 minutes post mixing, two replicate sets, (B) 30 minutes post mixing, two replicate sets. All proteins are at a final concentration of 10 µM. Scale bar is 10 µm.

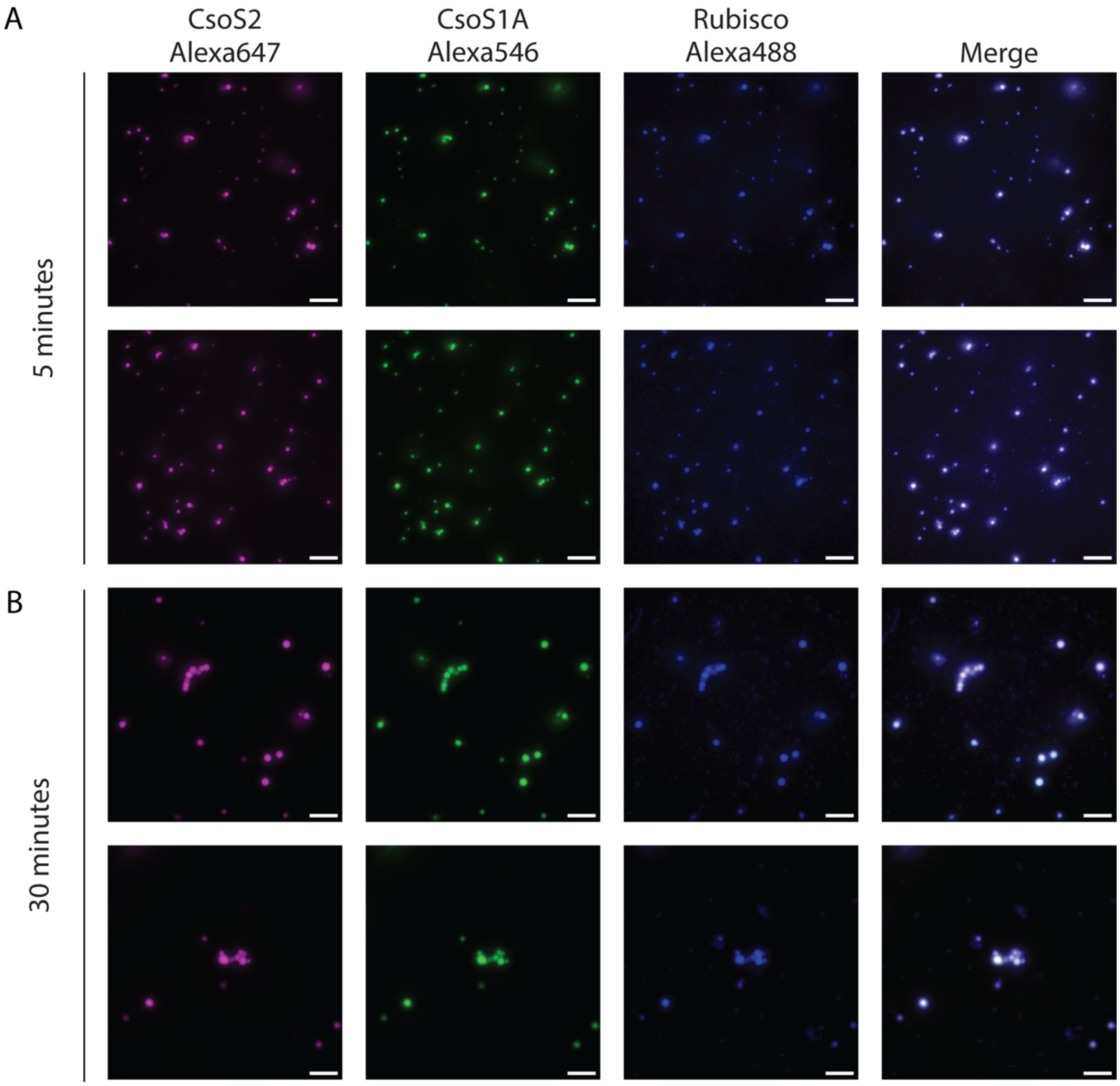

Figure S12. Rubisco, CsoS2, and CsoS1A form robust spherical condensates that grow in size over 30 minutes. CsoS2 is labeled in pink, shell (CsoS1A) in green, Rubisco in blue, and the merge appears as white. (A) 5 minutes post mixing, 2 replicate sets. (B) 30 minutes post mixing, 2 replicate sets. Final protein concentrations are 7.9 µM Rubisco, 6.1 µM CsoS2, and 17.5 µM shell. Scale bar is 10 µm.

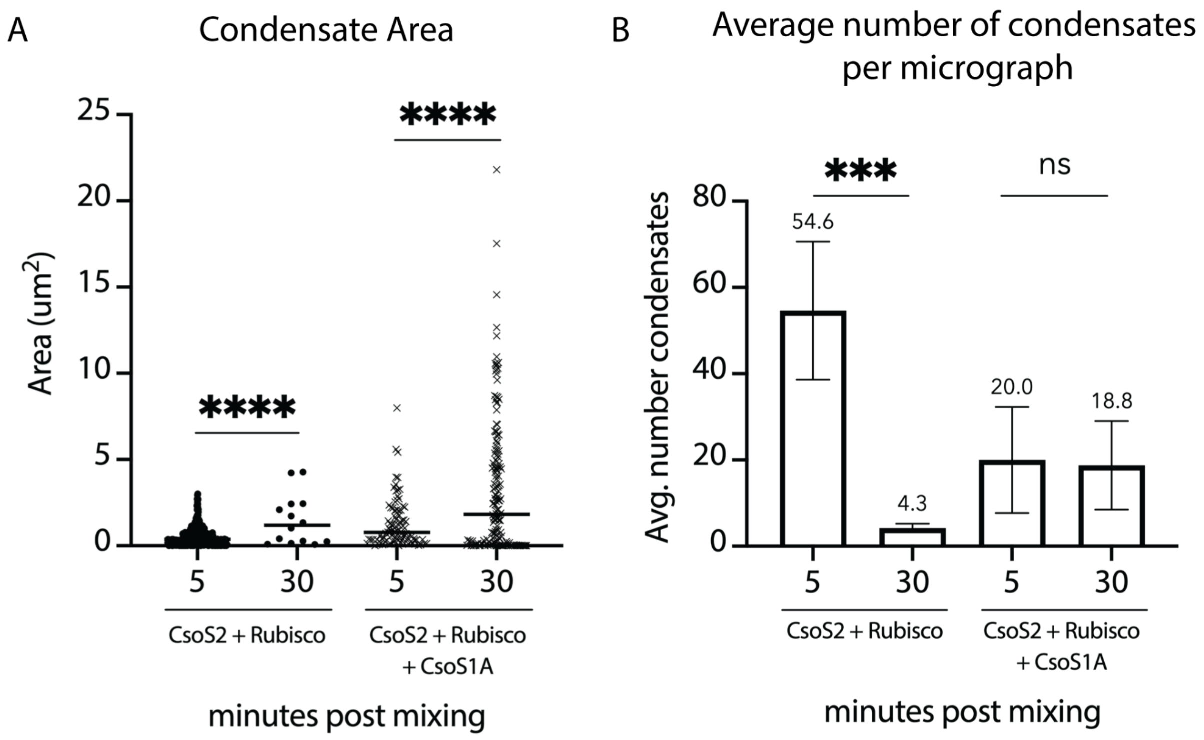

Figure S13. Addition of CsoS1A leads to an increase in droplet size. (A) Average area in µm^2 of CsoS2 + Rubisco condensates and CsoS2 + Rubisco + CsoS1A condensates at 5 minutes and 30 minutes post mixing. The median is indicated by a black line. Each individual droplet appears as a dot on the plot. Significance of **** is P ≤ 0.0001 in an unpaired t-test. (B) Average number of condensates per micrograph, which measured 83.2 x 83.2 µm. The average is written above each bar. Significance of *** is P ≤ 0.001 in an unpaired t-test. Ns, no significance.

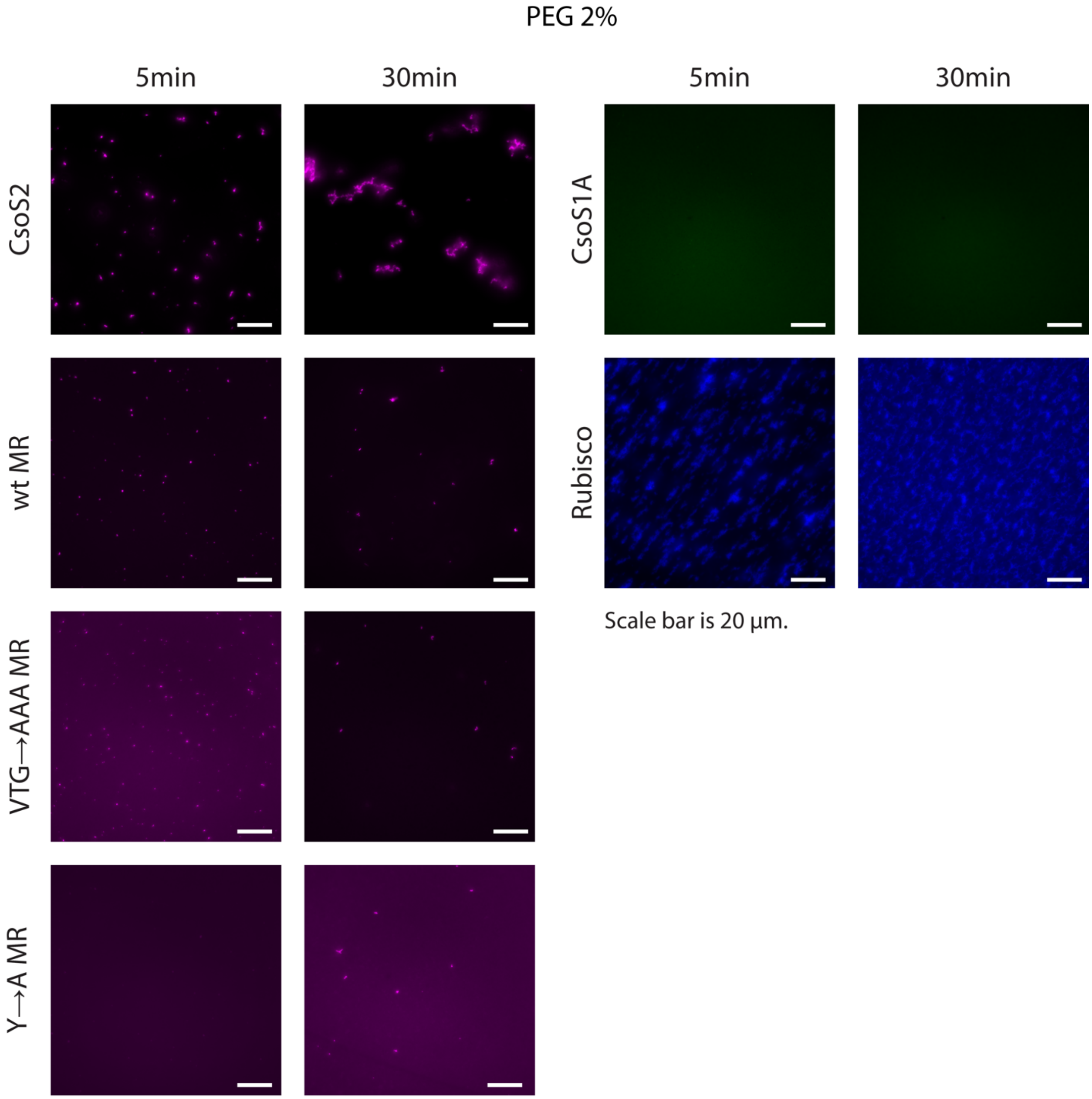

Figure S14. 2% PEG leads to condensate formation in some controls. CsoS2 and MR variants are shown in pink, CsoS1A is shown in green, and Rubisco is shown in blue. CsoS2 and MR variants were imaged on the Alexa-647 channel, CsoS1A on the Alexa-546 channel, and Rubisco on the Alexa-488 channel. Images were taken at 5 minutes and 30 minutes post mixing. All proteins are at a final concentration of 10 µM with 2% PEG-6000. Scale bar is 20 µm.

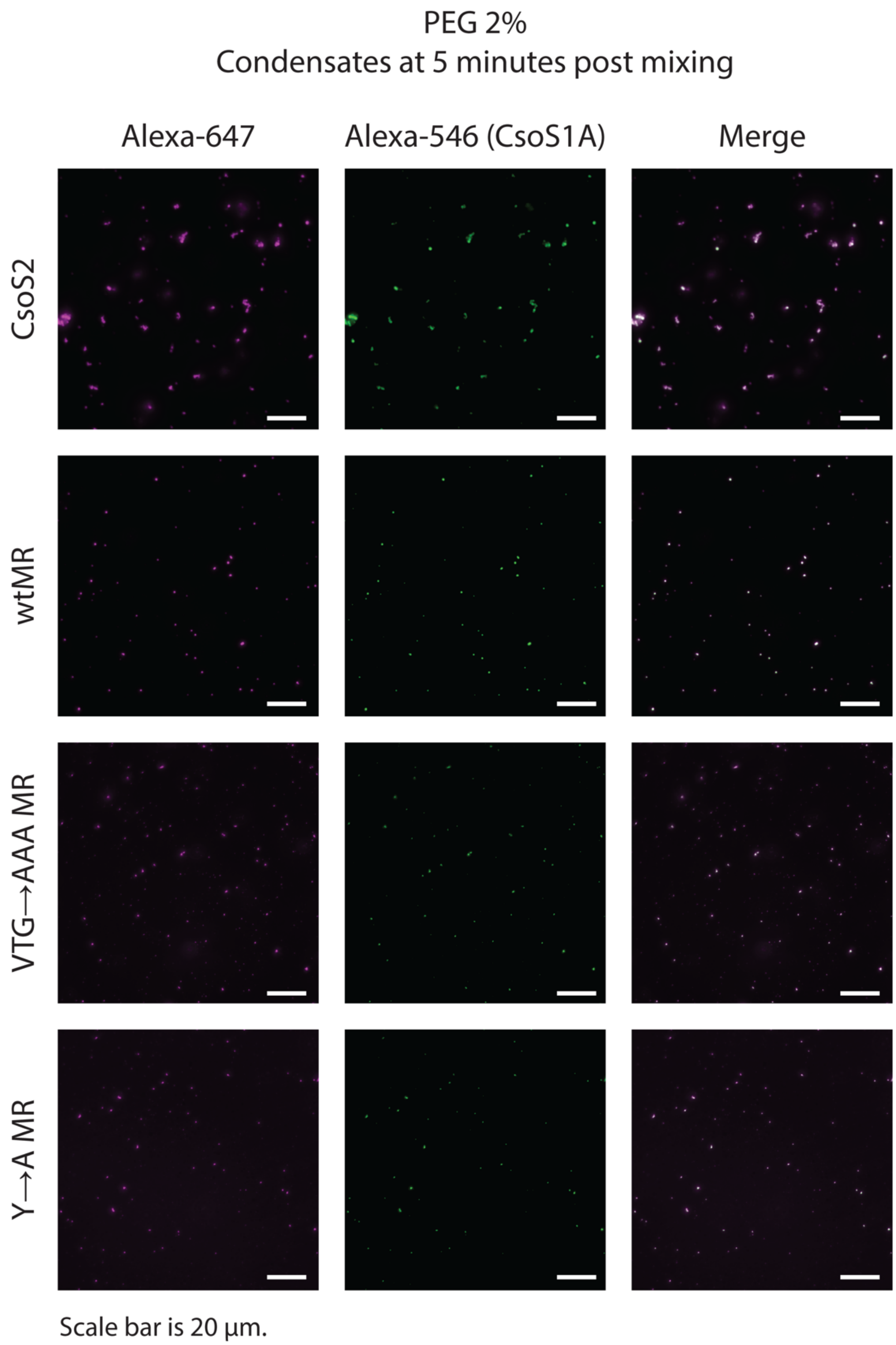

Figure S15. 2% PEG leads to some condensate formation of CsoS2 and MR variants with shell at 5 minutes post-mixing. Indicated CsoS2 / MR protein variants with added CsoS1A and 2% PEG-6000, imaged at 5 minutes post mixing. All CsoS2 / MR variants are labeled in pink, CsoS1A is labeled in green, and the merge appears white at equally overlapping intensities. All proteins are at a final concentration of 10 μM. Scale bar is 20 μm.

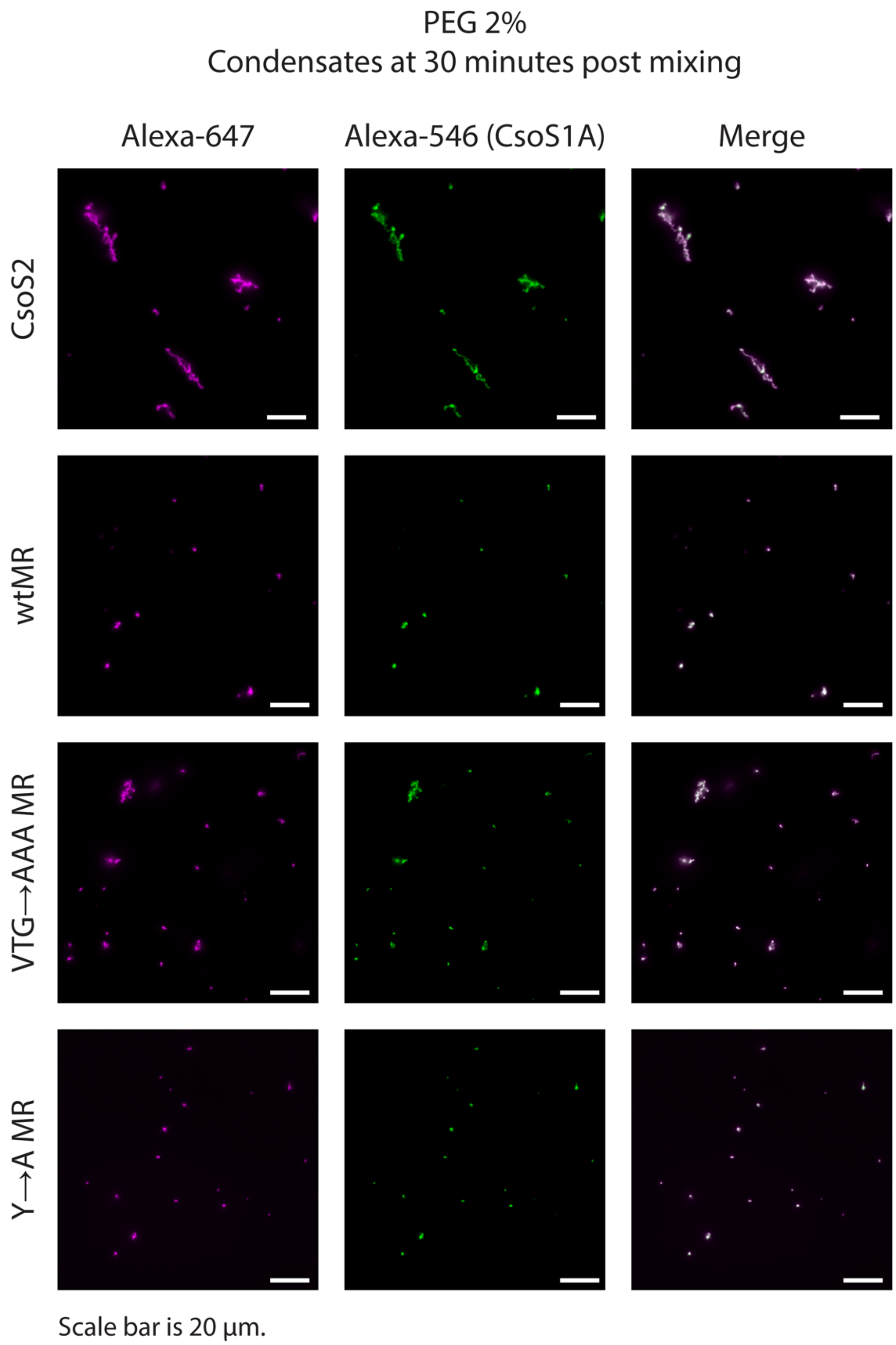

Figure S16. 2% PEG leads to some condensate formation of CsoS2 and MR variants with shell at 30 minutes post-mixing. Indicated CsoS2 / MR protein variants with added CsoS1A and 2% PEG-6000, imaged at 30 minutes post mixing. All CsoS2 / MR variants are labeled in pink, CsoS1A is labeled in green, and the merge appears white at equally overlapping intensities. All proteins are at a final concentration of 10 µM. Scale bar is 20 µm.

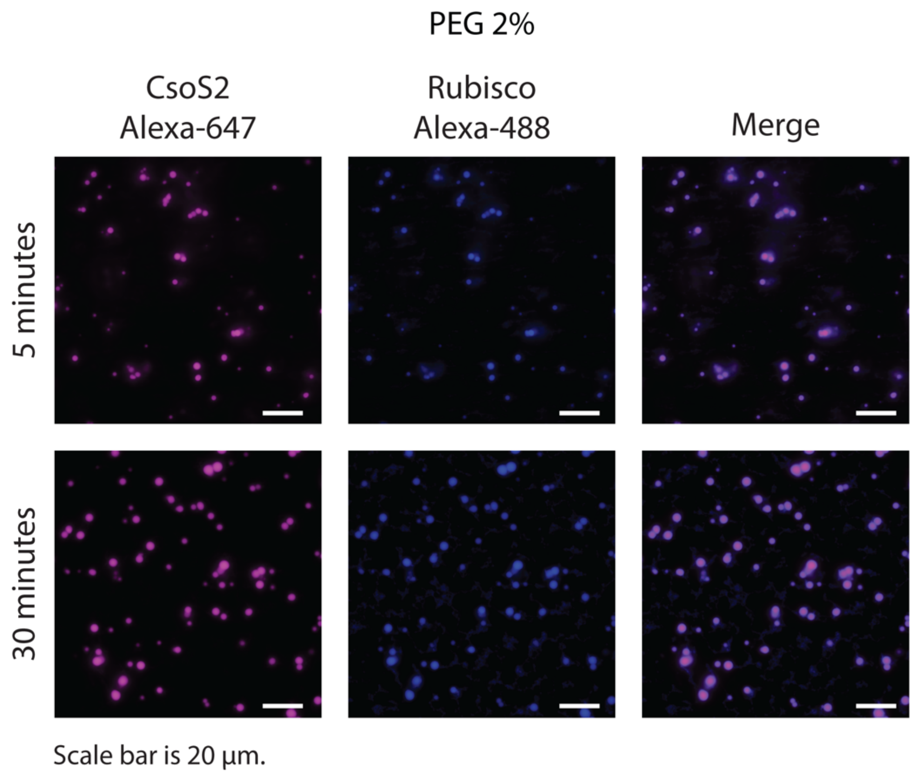

Figure S17. 2% PEG leads to CsoS2 and Rubisco condensate formation at 5 and 30 minutes post-mixing. CsoS2 and Rubisco with 2% PEG-6000. CsoS2 is labeled in pink, Rubisco is labeled in blue, and the merge appears purple at equally overlapping intensities. All proteins are at a final concentration of 10 µM. Scale bar is 20 µm.

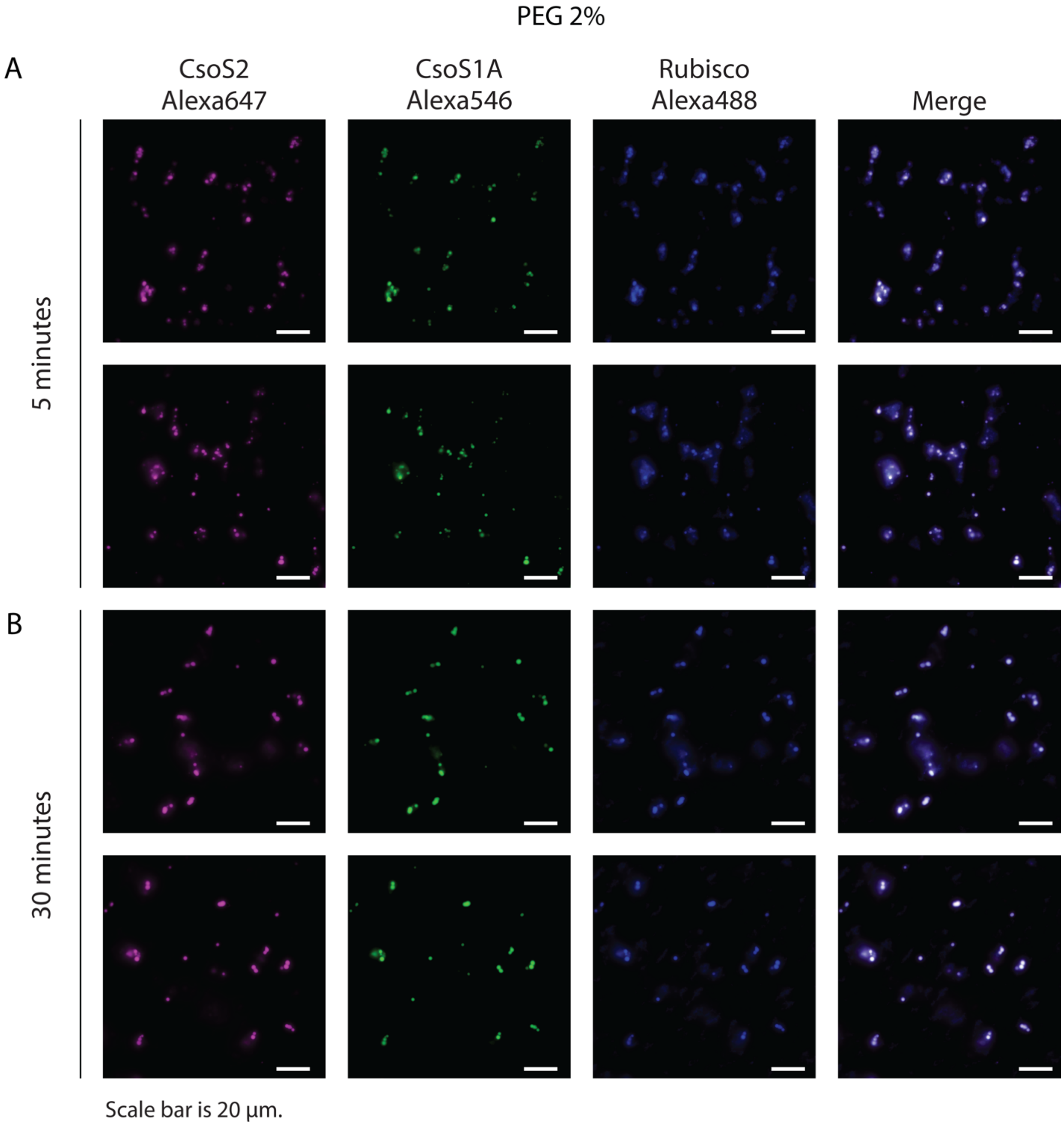

Figure S18. 2% PEG leads to Rubisco, CsoS2, and CsoS1A condensate formation at 5 and 30 minutes post-mixing. CsoS2 is labeled in pink, CsoS1A in green, Rubisco in blue, and the merge appears as white. (A) 5 minutes post mixing, 2 replicate sets. (B) 30 minutes post mixing, 2 replicate sets. Final protein concentrations are 7.9 µM Rubisco, 6.1 µM CsoS2, and 17.5 µM shell. Scale bar is 20 µm.

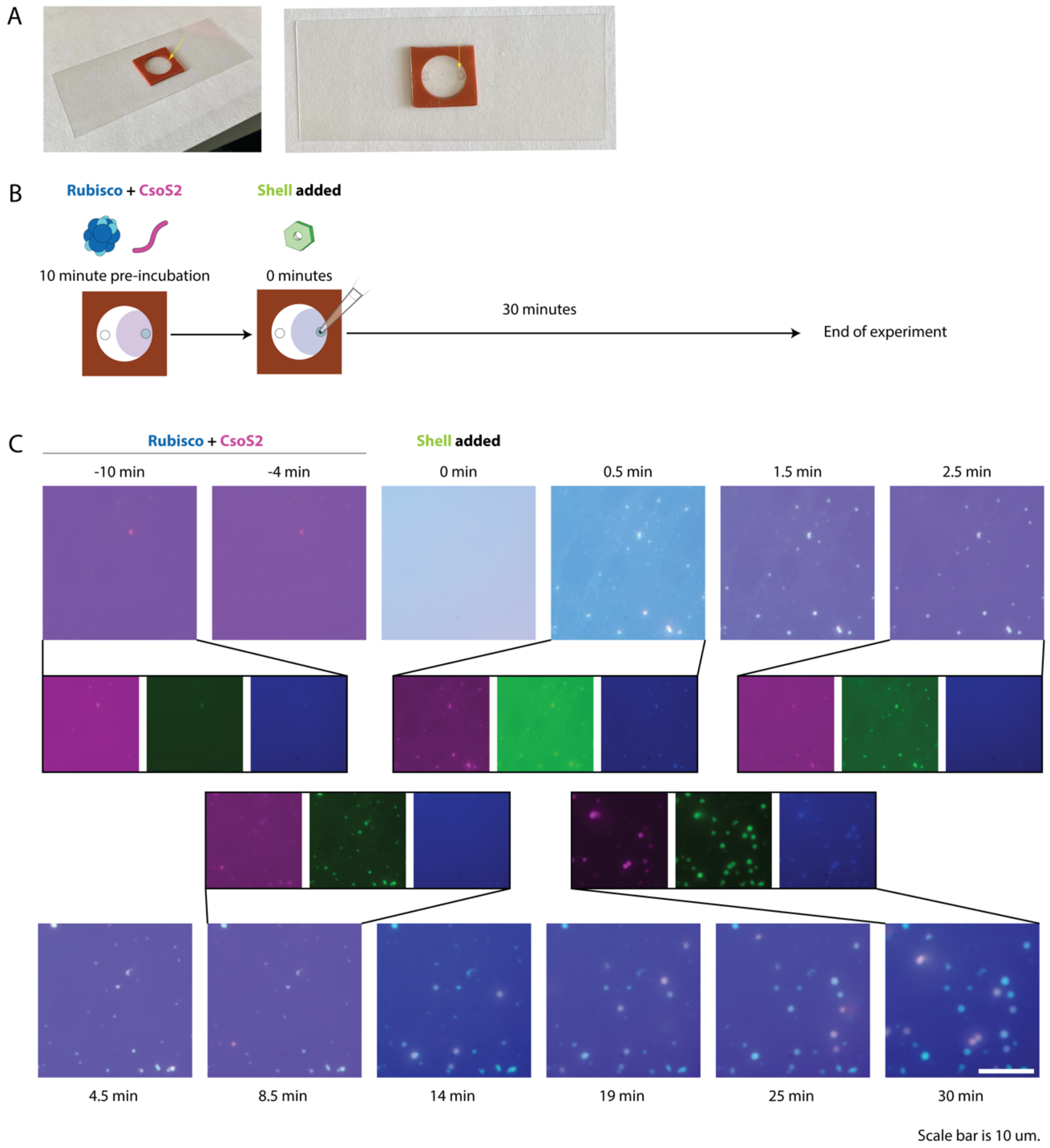

Figure S19. Adding shell to Rubisco + CsoS2 nucleates condensate formation. (A) Gasket setup; the gasket was affixed to a microscope slide, and protein solution was added with a pipette into the pore indicated by the yellow arrow. (B) Experimental schematic. Rubisco and CsoS2 were pre-incubated for 10 minutes before adding shell at time 0. Images were collected over 30 minutes. (C) Still micrographs from the addition experiment. Droplets appear in the focus plane as they condense and adhere to the slide. Larger micrographs show merged channels. Smaller micrographs from select timepoints show each individual channel: CsoS2 in pink, shell in green, and Rubisco in blue. Final concentrations are 8.3 µM Rubisco, 8.3 µM CsoS2, and 17.5 µM shell. Times were rounded to the nearest 30 seconds. Scale bar is 10 µm.

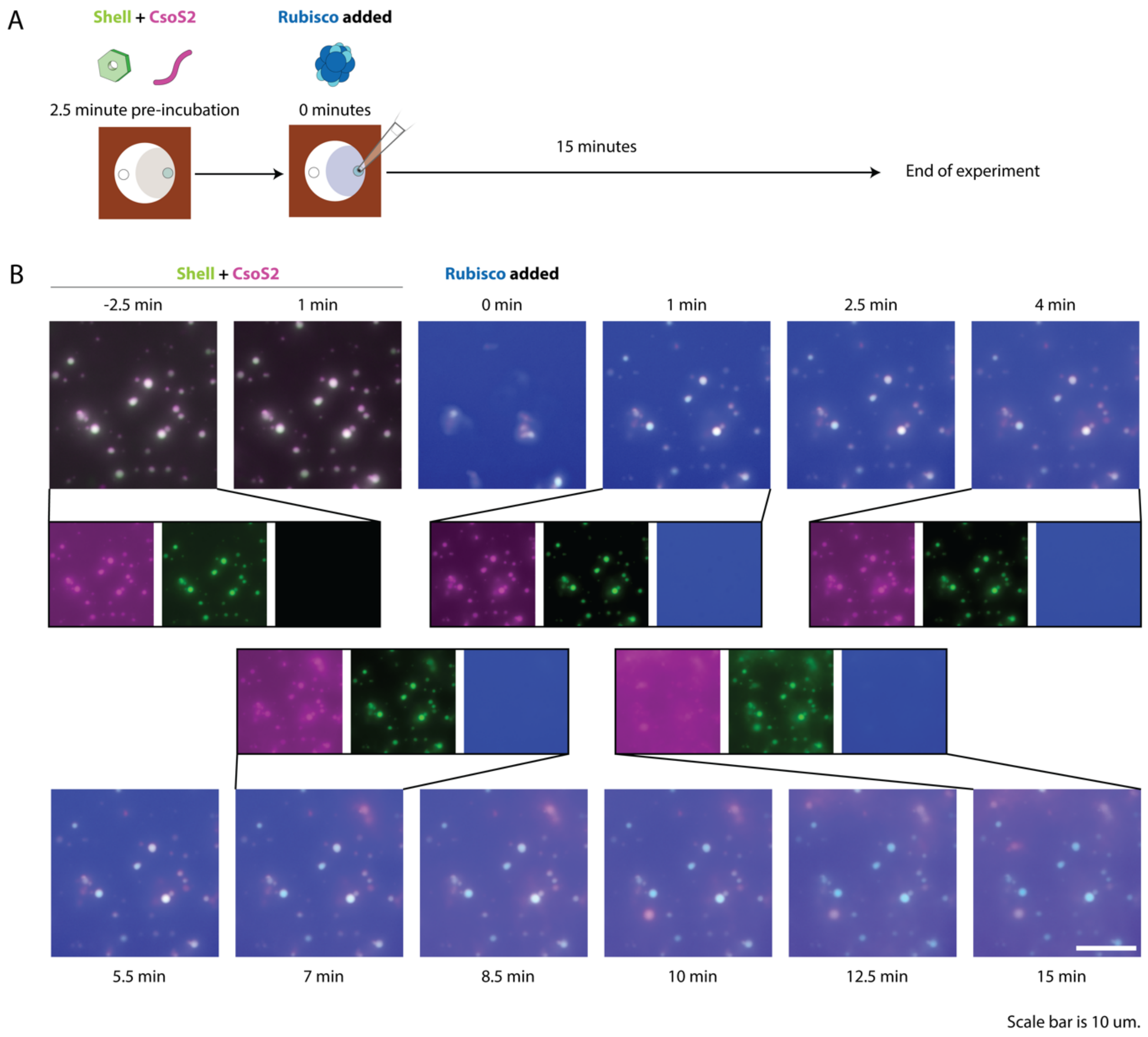

Figure S20. Shell + CsoS2 nucleates condensate formation, and Rubisco does not appear to add to condensates. (A) Experimental schematic. Shell and CsoS2 were pre-incubated for 2.5 minutes before adding Rubisco at time 0. Images were collected over 15 minutes. (B) Still micrographs from the addition experiment. Droplets appear in the focus plane as they condense and adhere to the slide. Larger micrographs show merged channels. Smaller micrographs from select timepoints show each individual channel: CsoS2 in pink, shell in green, and Rubisco in blue. Final concentrations are 10 µM Rubisco, 10 µM CsoS2, and 17.5 µM shell. Times were rounded to the nearest 30 seconds. Scale bar is 10 µm.

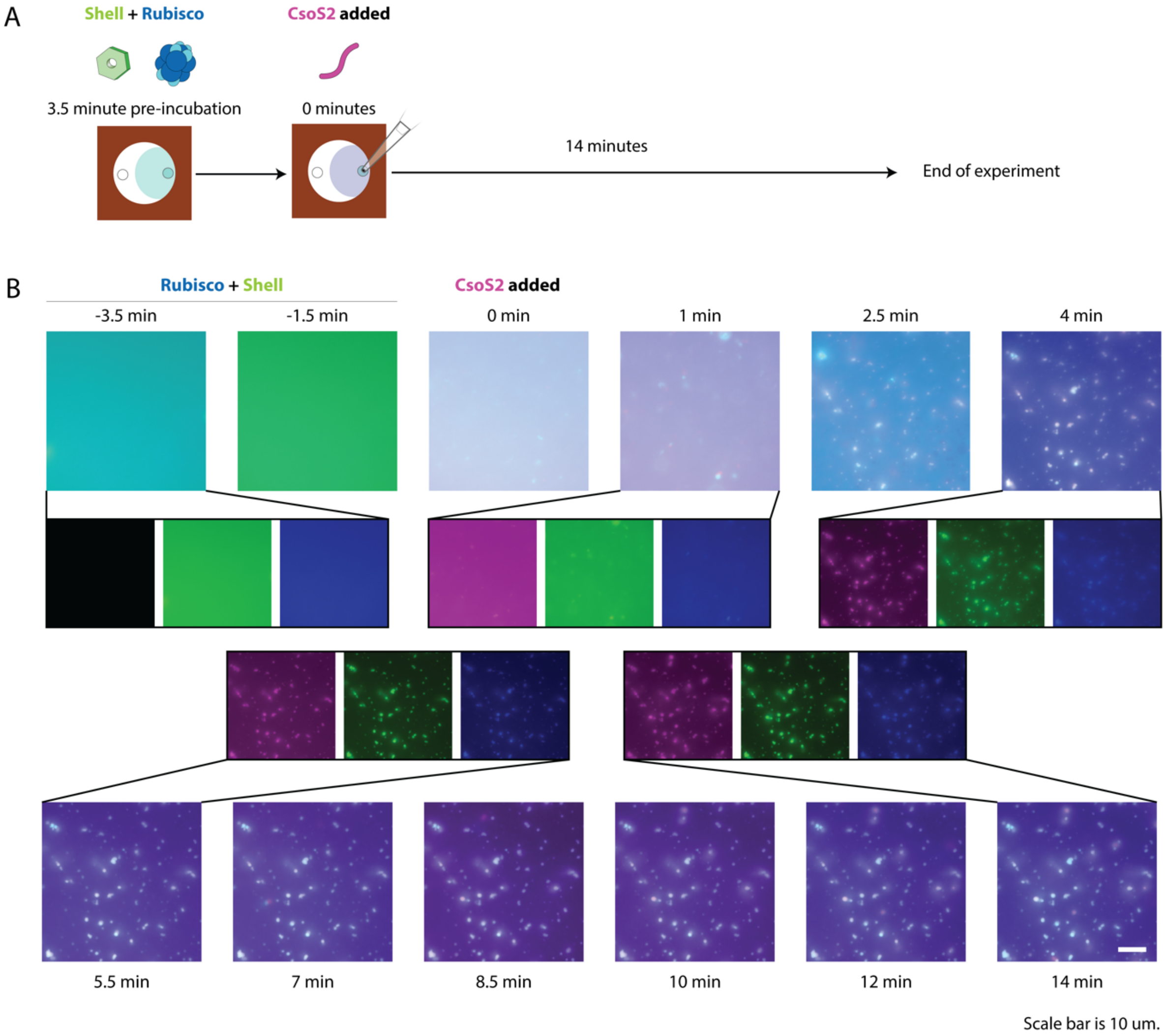

Figure S21. Adding CsoS2 to Rubisco + Shell nucleates condensate formation. (A) Experimental schematic. Shell and Rubisco were pre-incubated for 3.5 minutes before adding Rubisco at time 0. Images were collected over 14 minutes. (B) Still micrographs from the addition experiment. Droplets appear in the focus plane as they condense and adhere to the slide. Larger micrographs show merged channels. Smaller micrographs from select timepoints show each individual channel: CsoS2 in pink, shell in green, and Rubisco in blue. Final concentrations are 10 µM Rubisco, 10 µM CsoS2, and 17.5 µM shell. Times were rounded to the nearest 30 seconds. Scale bar is 10 µm.

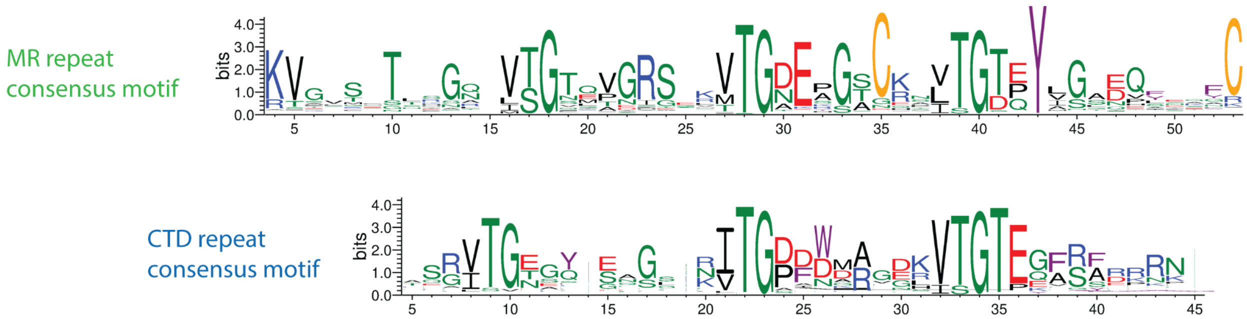

Figure S22. CTD repeat consensus motif. Comparison between the MR repeat consensus motif and the CTD repeat consensus motif. Sequence logo of the MR repeat was generated from an alignment of 1662 MR repeats identified across 272 dereplicated CsoS2 sequences. Sequence logo of the CTD repeat was generated from an alignment of 528 CTD repeats identified across the same 272 dereplicated CsoS2 sequences. Blue is basic, red is acidic, green is polar/small, black is hydrophobic, yellow is cysteine, purple is aromatic.

Table S1. Strains used in *H. neapolitanus* growth experiments.

H. neapolitanus strain	Plasmids used to generate strain	Genotype	Strain Resistance	Induction
N/A	pSAV108	Neutral site destination cloning vector, with the following features from 5'-3': *H. neapolitanus* upstream homology arm, KanR, LacIQ, pTRC promoter, BsaI site, LacZalpha, BsaI site, TrrnB terminator, *H. neapolitanus* downstream homology arm	kanamycin	N/A
WT	N/A	Halothiobacillus neapolitanus c2, ATCC 23641	N/A	N/A
ΔCsoS2	Ref. 24	Spectinomycin resistance cassette inserted into the native CsoS2 locus	spectinomycin	N/A
WT complement	pJB43	WT CsoS2, frameshift site intact	spectinomycin + kanamycin	IPTG
C→S MR1	pJB44	CsoS2 with C→S MR1 inserted into neutral site	spectinomycin + kanamycin	IPTG
C→S MR1-2	pJB45	CsoS2 with C→S CsoS2 MR1-2 inserted into neutral site	spectinomycin + kanamycin	IPTG
C→S MR1-4	pJB46	CsoS2 with C→S CsoS2 MR1-4 inserted into neutral site	spectinomycin + kanamycin	IPTG
C→S MR1-5	pJB47	CsoS2 with C→S CsoS2 MR1-5 inserted into neutral site	spectinomycin + kanamycin	IPTG
C→S MR1-6	pJB48	CsoS2 with C→S CsoS2 MR1-6 inserted into neutral site	spectinomycin + kanamycin	IPTG
VTG→AAA MR1	pJB51	CsoS2 with V(T/S)G→AAA CsoS2 MR1 inserted into neutral site	spectinomycin + kanamycin	IPTG
VTG→AAA MR1-3	pJB53	CsoS2 with V(T/S)G→AAA CsoS2 MR1-3 inserted into neutral site	spectinomycin + kanamycin	IPTG
VTG→AAA MR1-5	pJB55	CsoS2 with V(T/S)G→AAA CsoS2 MR1-5 inserted into neutral site	spectinomycin + kanamycin	IPTG
VTG→AAA MR1-7	pJB50	CsoS2 with V(T/S)G→AAA CsoS2 MR1-7 inserted into neutral site	spectinomycin + kanamycin	IPTG
Y→A MR1	pJB72	CsoS2 with Y→A CsoS2 MR1 inserted into neutral site	spectinomycin + kanamycin	IPTG
Y→A MR1-3	pJB73	CsoS2 with Y→A CsoS2 MR1-3 inserted into neutral site	spectinomycin + kanamycin	IPTG
Y→A MR1-5	pJB74	CsoS2 with Y→A CsoS2 MR1-5 inserted into neutral site	spectinomycin + kanamycin	IPTG
Y→A MR1-7	pJB68	CsoS2 with Y→A CsoS2 MR1-7 inserted into neutral site	spectinomycin + kanamycin	IPTG
K→A MR1-6	pJB67	CsoS2 with K→A CsoS2 MR1-6 inserted into neutral site	spectinomycin + kanamycin	IPTG

Table S2. Sequences used in this study.

Plasmid	Description	Resistance	MR Domain Sequence
	WT *H. neapolitanus* CsoS2 sequence (for reference)		(full CsoS2 sequence) Pink is NTD, Green is MR, and Blue is CTD<br>MSPSQSGMNPADLSGLSGKELARARRAALSKQGKAAVSNKTASVNRSTK<br>QAASSINTNQVRSSVNEVPTDYQMADQLCSTIDHADFGTESNRVRDLCRQ<br>RREALSTIGKKAVKTNGKPSGRVRPQQSVVHNDAMIENAGDTNQSSSTSL<br>NNELSEICSIADDMPERFGSQAKTVRDICRARRQALSERGTRAVPPKPQS<br>QGGPGRNGYQIDGYLDTALHGRDAAKRHREMLCQYGRGTAPSCKPTGR<br>VKNSVQSGNAAPKKVETGHTLSGGSVTGTQVDRKSHVTGNEPGTCRAVT<br>GTEYVGTEQFTSFCNTSPKPNATKVNVTTTARGRPVSGTEVSRTEKVTGN<br>ESGVCRNVTGTEYMSNEAHFSLCGTAAKPSQADKVMFGATARTHQVVSG<br>SDEFRPSSVTGNESGAKRTITGSQYADEGLARLTINGAPAKVARTHTFAGS<br>DVTGTEIGRSTRVTGDESGSCRSISGTEYLSNEQFQSFCDTKPQRSPFKV<br>GQDRTNKGQSVTGNLVDRSELVTGNEPGSCSRVTGSQYGQSKICGGGV<br>GKVRSMRTLRGTSVSGQQLDHAPKMSGDERGGCMPVTGNEYYGREHFE<br>PFCTSTPEPEAQSTEQSLTCEGQIISGTSVDASDLVTGNEIGEQQLISGDAY<br>VGAQQTGCLPTSPRFNQTGNVQSMGFKNTNQPEQNFAPGEVMPTDFSIQ<br>TPARSAQNRITGNDIAPSGRITGPGMLATGLITGTPEFRHAARELVGSPQP<br>MAMAMANRNKAAQAPVVQPEVVATQEKPELVCAPRSDQMDRVSGEGKE<br>RCHITGDDWSVNKHITGTAGQWASGRNPSMRGNARVVETSAFANRNVPK<br>PEKPGSKITGSSGNDTQGSLITYSGGARG*
pJB43	WT CsoS2, frameshift site intact	kanamycin	KVETGHTLSGGSVTGTQVDRKSHVTGNEPGTCRAVTGTEYVGTEQFTSF<br>CNTSPKPNATKVNVTTTARGRPVSGTEVSRTEKVTGNESGVCRNVTGTEY<br>MSNEAHFSLCGTAAKPSQADKVMFGATARTHQVVSGSDEFRPSSVTGNE<br>SGAKRTITGSQYADEGLARLTINGAPAKVARTHTFAGSDVTGTEIGRSTRV<br>TGDESGSCRSISGTEYLSNEQFQSFCDTKPQRSPFKVGQDRTNKGQSVT<br>GNLVDRSELVTGNEPGSCSRVTGSQYGQSKICGGGVGKVRSMRTLRGTS<br>VSGQQLDHAPKMSGDERGGCMPVTGNEYYGREHFEPFCTSTPEPEAQS<br>TEQSLTCEGQIISGTSVDASDLVTGNEIGEQQLISGDAYVGAQQTGCLPTS<br>PRFNQTGNVQSMGFKNTNQPEQN
pJB44	CsoS2 with C→S MR1	kanamycin	KVETGHTLSGGSVTGTQVDRKSHVTGNEPGTSRAVTGTEYVGTEQFTSF<br>SNTSPKPNATKVNVTTTARGRPVSGTEVSRTEKVTGNESGVCRNVTGTEY<br>MSNEAHFSLCGTAAKPSQADKVMFGATARTHQVVSGSDEFRPSSVTGNE<br>SGAKRTITGSQYADEGLARLTINGAPAKVARTHTFAGSDVTGTEIGRSTRV<br>TGDESGSCRSISGTEYLSNEQFQSFCDTKPQRSPFKVGQDRTNKGQSVT<br>GNLVDRSELVTGNEPGSCSRVTGSQYGQSKICGGGVGKVRSMRTLRGTS<br>VSGQQLDHAPKMSGDERGGCMPVTGNEYYGREHFEPFCTSTPEPEAQS<br>TEQSLTCEGQIISGTSVDASDLVTGNEIGEQQLISGDAYVGAQQTGCLPTS<br>PRFNQTGNVQSMGFKNTNQPEQN
pJB45	CsoS2 with C→S CsoS2 MR1-2	kanamycin	KVETGHTLSGGSVTGTQVDRKSHVTGNEPGTSRAVTGTEYVGTEQFTSF<br>SNTSPKPNATKVNVTTTARGRPVSGTEVSRTEKVTGNESGVSRNVTGTEY<br>MSNEAHFSLSGTAAKPSQADKVMFGATARTHQVVSGSDEFRPSSVTGNE<br>SGAKRTITGSQYADEGLARLTINGAPAKVARTHTFAGSDVTGTEIGRSTRV<br>TGDESGSCRSISGTEYLSNEQFQSFCDTKPQRSPFKVGQDRTNKGQSVT<br>GNLVDRSELVTGNEPGSCSRVTGSQYGQSKICGGGVGKVRSMRTLRGTS<br>VSGQQLDHAPKMSGDERGGCMPVTGNEYYGREHFEPFCTSTPEPEAQS<br>TEQSLTCEGQIISGTSVDASDLVTGNEIGEQQLISGDAYVGAQQTGCLPTS<br>PRFNQTGNVQSMGFKNTNQPEQN

pJB46	CsoS2 with C→S CsoS2 MR1-4	kanamycin	KVETGHTLSGGSVTGTQVDRKSHVTGNEPGTSRAVTGTEYVGTEQFTSF<br>SNTSPKPNATKVNVTTTARGRPVSGTEVSRTEKVTGNESGVSRNVTGTEY<br>MSNEAHFSLSGTAAKPSQADKVMFGATARTHQVVSGSDEFRPSSVTGNE<br>SGAKRTITGSQYADEGLARLTINGAPAKVARTHTFAGSDVTGTEIGRSTRV<br>TGDESGSSRSISGTEYLSNEQFQSFSDTKPQRSPFKVGQDRTNKGQSVT<br>GNLVDRSELVTGNEPGSCSRVTGSQYGQSKICGGGVGKVRSMRTLRGTS<br>VSGQQLDHAPKMSGDERGGCMPVTGNEYYGREHFEPFCTSTPEPEAQS<br>TEQSLTCEGQIISGTSVDASDLVTGNEIGEQQLISGDAYVGAQQTGCLPTS<br>PRFNQTGNVQSMGFKNTNQPEQN
pJB47	CsoS2 with C→S CsoS2 MR1-5	kanamycin	KVETGHTLSGGSVTGTQVDRKSHVTGNEPGTSRAVTGTEYVGTEQFTSF<br>SNTSPKPNATKVNVTTTARGRPVSGTEVSRTEKVTGNESGVSRNVTGTEY<br>MSNEAHFSLSGTAAKPSQADKVMFGATARTHQVVSGSDEFRPSSVTGNE<br>SGAKRTITGSQYADEGLARLTINGAPAKVARTHTFAGSDVTGTEIGRSTRV<br>TGDESGSSRSISGTEYLSNEQFQSFSDTKPQRSPFKVGQDRTNKGQSVT<br>GNLVDRSELVTGNEPGSSSRVTGSQYGQSKISGGGVGKVRSMRTLRGTS<br>VSGQQLDHAPKMSGDERGGCMPVTGNEYYGREHFEPFCTSTPEPEAQS<br>TEQSLTCEGQIISGTSVDASDLVTGNEIGEQQLISGDAYVGAQQTGCLPTS<br>PRFNQTGNVQSMGFKNTNQPEQN
pJB48	CsoS2 with C→S CsoS2 MR1-6	kanamycin	KVETGHTLSGGSVTGTQVDRKSHVTGNEPGTSRAVTGTEYVGTEQFTSF<br>SNTSPKPNATKVNVTTTARGRPVSGTEVSRTEKVTGNESGVSRNVTGTEY<br>MSNEAHFSLSGTAAKPSQADKVMFGATARTHQVVSGSDEFRPSSVTGNE<br>SGAKRTITGSQYADEGLARLTINGAPAKVARTHTFAGSDVTGTEIGRSTRV<br>TGDESGSSRSISGTEYLSNEQFQSFSDTKPQRSPFKVGQDRTNKGQSVT<br>GNLVDRSELVTGNEPGSSSRVTGSQYGQSKISGGGVGKVRSMRTLRGTS<br>VSGQQLDHAPKMSGDERGGSMPVTGNEYYGREHFEPFSTSTPEPEAQST<br>EQSLTCEGQIISGTSVDASDLVTGNEIGEQQLISGDAYVGAQQTGCLPTSP<br>RFNQTGNVQSMGFKNTNQPEQN
pJB51	CsoS2 with V(T/S)G→AAA CsoS2 MR1	kanamycin	KVETGHTLSGGSAAATQVDRKSHAAANEPGTCRAAAATEYVGTEQFTSFC<br>NTSPKPNATKVNVTTTARGRPVSGTEVSRTEKVTGNESGVCRNVTGTEY<br>MSNEAHFSLCGTAAKPSQADKVMFGATARTHQVVSGSDEFRPSSVTGNE<br>SGAKRTITGSQYADEGLARLTINGAPAKVARTHTFAGSDVTGTEIGRSTRV<br>TGDESGSCRSISGTEYLSNEQFQSFCDTKPQRSPFKVGQDRTNKGQSVT<br>GNLVDRSELVTGNEPGSCSRVTGSQYGQSKICGGGVGKVRSMRTLRGTS<br>VSGQQLDHAPKMSGDERGGCMPVTGNEYYGREHFEPFCTSTPEPEAQS<br>TEQSLTCEGQIISGTSVDASDLVTGNEIGEQQLISGDAYVGAQQTGCLPTS<br>PRFNQTGNVQSMGFKNTNQPEQN
pJB53	CsoS2 with V(T/S)G→AAA CsoS2 MR1-3	kanamycin	KVETGHTLSGGSAAATQVDRKSHAAANEPGTCRAAAATEYVGTEQFTSFC<br>NTSPKPNATKVNVTTTARGRPAAATEVSRTEKAAANESGVCRNAAATEYM<br>SNEAHFSLCGTAAKPSQADKVMFGATARTHQVAAASDEFRPSSAAANES<br>GAKRTITGSQYADEGLARLTINGAPAKVARTHTFAGSDVTGTEIGRSTRVT<br>GDESGSCRSISGTEYLSNEQFQSFCDTKPQRSPFKVGQDRTNKGQSVTG<br>NLVDRSELVTGNEPGSCSRVTGSQYGQSKICGGGVGKVRSMRTLRGTSV<br>SGQQLDHAPKMSGDERGGCMPVTGNEYYGREHFEPFCTSTPEPEAQST<br>EQSLTCEGQIISGTSVDASDLVTGNEIGEQQLISGDAYVGAQQTGCLPTSP<br>RFNQTGNVQSMGFKNTNQPEQN

pJB55	CsoS2 with<br>V(T/S)G→AA<br>A CsoS2<br>MR1-5	kanamycin	KVETGHTLSGGSAAATQVDRKSHAAANEPGTCRAAAATEYVGTEQFTSFCNTSPKPNATKVNVTTTARGRPAAATEVSRTEKAAANESGVCRNAAATEYMSNEAHFSLCGTAAKPSQADKVMFGATARTHQVAAASDEFRPSSAAANESGAKRTITGSQYADEGLARLTINGAPAKVARTHTFAGSDAAATEIGRSTRAAADESGSCRSISGTEYLSNEQFQSFCDTKPQRSPFKVGQDRTNKGQSAAANLVDRSELAAANEPGSCSRAAASQYGQSKICGGGVGKVRSMRTLRGTSVSGQQLDHAPKMSGDERGGCMPVTGNEYYGREHFEPFCTSTPEPEAQSTEQSLTCEGQIISGTSVDASDLVTGNEIGEQQLISGDAYVGAQQTGCLPTSPRFNQTGNVQSMGFKNTNQPEQN
pJB50	CsoS2 with<br>V(T/S)G→AA<br>A CsoS2<br>MR1-7	kanamycin	KVETGHTLSGGSAAATQVDRKSHAAANEPGTCRAAAATEYVGTEQFTSFCNTSPKPNATKVNVTTTARGRPAAATEVSRTEKAAANESGVCRNAAATEYMSNEAHFSLCGTAAKPSQADKVMFGATARTHQVAAASDEFRPSSAAANESGAKRTITGSQYADEGLARLTINGAPAKVARTHTFAGSDAAATEIGRSTRAAADESGSCRSISGTEYLSNEQFQSFCDTKPQRSPFKVGQDRTNKGQSAAANLVDRSELAAANEPGSCSRAAASQYGQSKICGGGVGKVRSMRTLRGTSAAAQQLDHAPKMSGDERGGCMPAAANEYYGREHFEPFCTSTPEPEAQSTEQSLTCEGQIISGTSVDASDLAAANEIGEQQLISGDAYVGAQQTGCLPTSPRFNQTGNVQSMGFKNTNQPEQN
pJB72	CsoS2 with<br>Y→A CsoS2<br>MR1	kanamycin	KVETGHTLSGGSVTGTQVDRKSHVTGNEPGTCRAVTGTEAVGTEQFTSFCNTSPKPNATKVNVTTTARGRPVSGTEVSRTEKVTGNESGVCRNVTGTEYMSNEAHFSLCGTAAKPSQADKVMFGATARTHQVVSGSDEFRPSSVTGNESGAKRTITGSQYADEGLARLTINGAPAKVARTHTFAGSDVTGTEIGRSTRVTGDESGSCRSISGTEYLSNEQFQSFCDTKPQRSPFKVGQDRTNKGQSVTGNLVDRSELVTGNEPGSCSRVTGSQYGQSKICGGGVGKVRSMRTLRGTSVSGQQLDHAPKMSGDERGGCMPVTGNEYYGREHFEPFCTSTPEPEAQSTEQSLTCEGQIISGTSVDASDLVTGNEIGEQQLISGDAYVGAQQTGCLPTSPRFNQTGNVQSMGFKNTNQPEQN
pJB73	CsoS2 with<br>Y→A CsoS2<br>MR1-3	kanamycin	KVETGHTLSGGSVTGTQVDRKSHVTGNEPGTCRAVTGTEAVGTEQFTSFCNTSPKPNATKVNVTTTARGRPVSGTEVSRTEKVTGNESGVCRNVTGTEAMSNEAHFSLCGTAAKPSQADKVMFGATARTHQVVSGSDEFRPSSVTGNESGAKRTITGSQAADEGLARLTINGAPAKVARTHTFAGSDVTGTEIGRSTRVTGDESGSCRSISGTEYLSNEQFQSFCDTKPQRSPFKVGQDRTNKGQSVTGNLVDRSELVTGNEPGSCSRVTGSQYGQSKICGGGVGKVRSMRTLRGTSVSGQQLDHAPKMSGDERGGCMPVTGNEYYGREHFEPFCTSTPEPEAQSTEQSLTCEGQIISGTSVDASDLVTGNEIGEQQLISGDAYVGAQQTGCLPTSPRFNQTGNVQSMGFKNTNQPEQN
pJB74	CsoS2 with<br>Y→A CsoS2<br>MR1-5	kanamycin	KVETGHTLSGGSVTGTQVDRKSHVTGNEPGTCRAVTGTEAVGTEQFTSFCNTSPKPNATKVNVTTTARGRPVSGTEVSRTEKVTGNESGVCRNVTGTEAMSNEAHFSLCGTAAKPSQADKVMFGATARTHQVVSGSDEFRPSSVTGNESGAKRTITGSQAADEGLARLTINGAPAKVARTHTFAGSDVTGTEIGRSTRVTGDESGSCRSISGTEALSNEQFQSFCDTKPQRSPFKVGQDRTNKGQSVTGNLVDRSELVTGNEPGSCSRVTGSQAGQSKICGGGVGKVRSMRTLRGTSVSGQQLDHAPKMSGDERGGCMPVTGNEYYGREHFEPFCTSTPEPEAQSTEQSLTCEGQIISGTSVDASDLVTGNEIGEQQLISGDAYVGAQQTGCLPTSPRFNQTGNVQSMGFKNTNQPEQN

pJB68	CsoS2 with Y→A CsoS2 MR1-7	kanamycin	KVETGHTLSGGSVTGTQVDRKSHVTGNEPGTCRAVTGTEAVGTEQFTSF CNTSPKPNATKVNVTTTARGRPVSGTEVSRTEKVTGNESGVCRNVTGTEA MSNEAHFSLCGTAAKPSQADKVMFGATARTHQVVSGSDEFRPSSVTGNE SGAKRTITGSQAADEGLARLTINGAPAKVARTHTFAGSDVTGTEIGRSTRV TGDESGSCRSISGTEALSNEQFQSFCDTKPQRSPFKVGQDRTNKGQSVT GNLVDRSELVTGNEPGSCSRVTGSQAGQSKICGGGVGKVRSMRTLRGTS VSGQQLDHAPKMSGDERGGCMPVTGNEAYGREHFEPFCTSTPEPEAQS TEQSLTCEGQIISGTSVDASDLVTGNEIGEQQLISGDAAVGAQQTGCLPTS PRFNQTGNVQSMGFKNTNQPEQN
pJB67	CsoS2 with K→A CsoS2 MR1-6	kanamycin	AVETGHTLSGGSVTGTQVDRKSHVTGNEPGTCRAVTGTEYVGTEQFTSF CNTSPKPNATAVNVTTTARGRPVSGTEVSRTEKVTGNESGVCRNVTGTEY MSNEAHFSLCGTAAKPSQADAVMFGATARTHQVVSGSDEFRPSSVTGNE SGAKRTITGSQYADEGLARLTINGAPAAVARTHTFAGSDVTGTEIGRSTRV TGDESGSCRSISGTEYLSNEQFQSFCDTKPQRSPFAVGQDRTNKGQSVT GNLVDRSELVTGNEPGSCSRVTGSQYGQSKICGGGVGAVRSMRTLRGTS VSGQQLDHAPKMSGDERGGCMPVTGNEYYGREHFEPFCTSTPEPEAQS TEQSLTCEGQIISGTSVDASDLVTGNEIGEQQLISGDAYVGAQQTGCLPTS PRFNQTGNVQSMGFKNTNQPEQN
pLz92	6xHis-CsoS2B	carbenicillin	MHHHHHHPSQSGMNPADLSGLSGKELARARRAALSKQGKAAVSNKTASV NRSTKQAASSINTNQVRSSVNEVPTDYQMADQLCSTIDHADFGTESNRVR DLCRQRREALSTIGKKAVKTNGKPSGRVRPQQSVVHNDAMIENAGDTNQ SSSTSLNNELSEICSIADDMPERFGSQAKTVRDICRARRQALSERGTRAVP PKPQSQGGPGRNGYQIDGYLDTALHGRDAAKRHREMLCQYGRGTAPSC KPTGRVKNSVQSGNAAPKKVETGHTLSGGSVTGTQVDRKSHVTGNEPGT CRAVTGTEYVGTEQFTSFCNTSPKPNATKVNVTTTARGRPVSGTEVSRTE KVTGNESGVCRNVTGTEYMSNEAHFSLCGTAAKPSQADKVMFGATARTH QVVSGSDEFRPSSVTGNESGAKRTITGSQYADEGLARLTINGAPAKVART HTFAGSDVTGTEIGRSTRVTGDESGSCRSISGTEYLSNEQFQSFCDTKPQ RSPFKVGQDRTNKGQSVTGNLVDRSELVTGNEPGSCSRVTGSQYGQSKI CGGGVGKVRSMRTLRGTSVSGQQLDHAPKMSGDERGGCMPVTGNEYY GREHFEPFCTSTPEPEAQSTEQSLTCEGQIISGTSVDASDLVTGNEIGEQQ LISGDAYVGAQQTGCLPTSPRFNQTGNVQSMGFKNTNQPEQNFAPGEVM PTDFSIQTPARSAQNRITGNDIAPSGRITGPGMLATGLITGTPEFRHAAREL VGSPQPMAMAMANRNKAAQAPVVQPEVVATQEKPELVCAPRSDQMDRV SGEGKERCHITGDDWSVNKHITGTAGQWASGRNPSMRGNARVVETSAFA NRNVPKPEKPGSKITGSSGNDTQGSLITYSGGARG*
pBz84	6xHis-(wtMR1-6)-strep	carbenicillin	MSHHHHHHAPKKVETGHTLSGGSVTGTQVDRKSHVTGNEPGTCRAVTGT EYVGTEQFTSFCNTSPKPNATKVNVTTTARGRPVSGTEVSRTEKVTGNES GVCRNVTGTEYMSNEAHFSLCGTAAKPSQADKVMFGATARTHQVVSGSD EFRPSSVTGNESGAKRTITGSQYADEGLARLTINGAPAKVARTHTFAGSDV TGTEIGRSTRVTGDESGSCRSISGTEYLSNEQFQSFCDTKPQRSPFKVGQ DRTNKGQSVTGNLVDRSELVTGNEPGSCSRVTGSQYGQSKICGGGVGKV RSMRTLRGTSVSGQQLDHAPKMSGDERGGCMPVTGNEYYGREHFEPFC TSTPEPEAQWSHPQFEK*
pJB65	6xHis-(VTG→AAA MR1-6)-strep	carbenicillin	MSHHHHHHAPKKVETGHTLSGGSAAATQVDRKSHAAANEPGTCRAAAAT EYVGTEQFTSFCNTSPKPNATKVNVTTTARGRPAAATEVSRTEKAAANES GVCRNAAATEYMSNEAHFSLCGTAAKPSQADKVMFGATARTHQVAAASD EFRPSSAAANESGAKRTITGSQYADEGLARLTINGAPAKVARTHTFAGSDA AATEIGRSTRAAADESGSCRSISGTEYLSNEQFQSFCDTKPQRSPFKVGQ DRTNKGQSAAANLVDRSELAAANEPGSCSRAAASQYGQSKICGGGVGKV

			RSMRTLRGTSVSGQQLDHAPKMSGDERGGCMPVTGNEYYGREHFEPFC<br>TSTPEPEAQWSHPQFEK*
pJB75	6xHis-(Y→A MR1-6)-strep	carbenicillin	MSHHHHHHAPKKVETGHTLSGGSVTGTQVDRKSHVTGNEPGTCRAVTGT<br>EAVGTEQFTSFCNTSPKPNATKVNVTTTARGRPVSGTEVSRTEKVTGNES<br>GVCRNVTGTEAMSNEAHFSLCGTAAKPSQADKVMFGATARTHQVVSGSD<br>EFRPSSVTGNESGAKRTITGSQAADEGLARLTINGAPAKVARTHTFAGSDV<br>TGTEIGRSTRVTGDESGSCRSISGTEALSNEQFQSFCDTKPQRSPFKVGQ<br>DRTNKGQSVTGNLVDRSELVTGNEPGSCSRVTGSQAGQSKICGGGVGKV<br>RSMRTLRGTSVSGQQLDHAPKMSGDERGGCMPVTGNEAYGREHFEPFC<br>TSTPEPEAQWSHPQFEK*
pBz12	6xHis-Rubisco	carbenicillin	<u>CbbL</u><br>MSAVKKYSAGVKEYRQTYWMPEYTPLDSDILACFKITPQPGVDREEAAAA<br>VAAESSTGTWTTVWTDLLTDMDYYKGRAYRIEDVPGDDAAFYAFIAYPIDL<br>FEEGSVVNVFTSLVGNVFGFKAVRGLRLEDVRFPLAYVKTCGGPPHGIQV<br>ERDKMNKYGRPLLGCTIKPKLGLSAKNYGRAVYECLRGGLDFTKDDENIN<br>SQPFMRWRDRFLFVQDATETAEAQTGERKGHYLNVTAPTPEEMYKRAEF<br>AKEIGAPIIMHDYITGGFTANTGLAKWCQDNGVLLHIHRAMHAVIDRNPNH<br>GIHFRVLTKILRLSGGDHLHTGTVVGKLEGDRASTLGWIDLLRESFIPEDRS<br>RGIFFDQDWGSMPGVFAVASGGIHVWHMPALVNIFGDDSVLQFGGGTLG<br>HPWGNAAGAAANRVALEACVEARNQGRDIEKEGKEILTAAAQHSPELKIA<br>METWKEIKFEFDTVDKLDTQNRHHHHHH*<br><u>CbbS</u><br>MAEMQDYKQSLKYETFSYLPPMNAERIRAQIKYAIAQGWSPGIEHVEVKNS<br>MNQYWYMWKLPFFGEQNVDNVLAEIEACRSAYPTHQVKLVAYDNYAQSL<br>GLAFVVYRGN*
pLz48	strep-CsoS1A	carbenicillin	MSWSHPQFEKENLYFQSADVTGIALGMIETRGLVPAIEAADAMTKAAEVRL<br>VGRQFVGGGYVTVLVRGETGAVNAAVRAGADACERVGDGLVAAHIIARVH<br>SEVENILPKAPQA*
